# Lipid-induced S-palmitoylation of Insulin-Responsive Aminopeptidase (IRAP) drives the onset of insulin resistance in the heart

**DOI:** 10.1007/s00018-026-06179-0

**Published:** 2026-03-26

**Authors:** Francesco Schianchi, Jeroen Guns, Freek G. Bouwman, Jeroen F.J. Bogie, Miranda Nabben, Agnieszka Strzelecka, Rick van Leeuwen, Dimitris Kapsokalyvas, Kaitlyn M.J.H. Dennis, Lisa C. Heather, Shujin Wang, Jan F.C. Glatz, Dietbert Neumann, Joost J.F.P. Luiken

**Affiliations:** 1 Department of Genetics & Cell Biology, Maastricht University, Maastricht, 6229 ER The Netherlands; 2 Department of Cardiology, Maastricht University Medical Center+, Maastricht, The Netherlands; 3 CARIM School for Cardiovascular Diseases, Maastricht, The Netherlands; 4 Department of Immunology and Infection, Biomedical Research Institute, Hasselt University, Hasselt, Belgium; 5 Department of Human Biology, NUTRIM School of Nutrition and Translational Research in Metabolism, Faculty of Health, Medicine and Life Sciences, Maastricht, The Netherlands; 6 Department of Clinical Genetics, Maastricht University Medical Center+, Maastricht, The Netherlands; 7 Interdisciplinary Centre for Clinical Research IZKF, University Hospital RWTH Aachen, Aachen, Germany; 8 Department of Physiology, Anatomy and Genetics, University of Oxford, Oxford, UK; 9 Institute of Life Sciences, Chongqing Medical University, Chongqing, PR China; 10 Departments of Pathology, CARIM School for Cardiovascular Diseases, Maastricht University, Maastricht, The Netherlands

**Keywords:** Cardiac lipid-induced insulin resistance, S-palmitoylation, DHHC, Insulin-stimulated glucose uptake, IRAP

## Abstract

Excessive accumulation of long-chain fatty acids (LCFAs) in cardiomyocytes leads to cardiac lipid-induced insulin resistance (CLIR), impairing insulin signaling and glucose uptake. This metabolic disruption marks a prediabetic state in which cardiomyocytes rely predominantly on lipids for energy provision, leading to lipotoxicity and reduced contractile function over time. Palmitate, the most common dietary LCFA, also serves as a substrate for protein S-palmitoylation, a reversible lipid-based post-translational modification (PTM) that governs protein localization and trafficking through distinct palmitoyl acyltransferase (PATs) isoforms, also denominated as DHHC. In this study, we demonstrate that pharmacological inhibition of protein S-palmitoylation with 2-bromopalmitate (2BP) prevents contractile dysfunction and CLIR in palmitate-overloaded cardiomyocytes. Analysis of the cardiomyocyte palmitoylome disclosed, among other proteins, the insulin-regulated aminopeptidase (IRAP). IRAP translocates together with glucose transporter GLUT4 to the plasma membrane in response to insulin. LCFA-induced hyper-S-palmitoylation of IRAP, mediated by DHHC5, disrupts glucose uptake. Knockdown of DHHC5 or transduction with S-palmitoylation-deficient IRAP rescues both cardiomyocyte glucose uptake and contractile function. These findings identify protein S-palmitoylation, particularly IRAP hyper-S-palmitoylation, as a novel driver of CLIR and associated myocardial dysfunction.

**Supplementary Information:**

The online version contains supplementary material available at 10.1007/s00018-026-06179-0.

## Introduction

The heart primarily relies on long-chain fatty acids (LCFAs) and glucose for sustaining contractile activity [1]. An optimal balance of ~ 60% and ~ 40%, respectively, in their contribution to ATP production is strongly associated with cardiac health. Deviation from this optimal balance is seen as a causal factor leading to cardiac diseases [2, 3]. The cardiac substrate balance is mainly determined at the level of plasmalemmal uptake of both substrates by their respective transporters, representing the rate-limiting step in cardiac metabolism. CD36 is the main cardiac LCFAs transporter, whereas glucose uptake is mediated by GLUT1 and GLUT4 [4]. GLUT1 resides predominantly at the plasma membrane and mediates a basal influx of glucose into cardiomyocytes [5, 6]. In contrast, GLUT4 is mainly sequestered within intracellular GLUT4 Storage Vesicles (GSVs). Upon stimulation by insulin, the GSVs are translocated to the plasma membrane, thereby increasing cell surface exposure of GLUT4 and its trafficking companion Insulin-responsive aminopeptidase (IRAP) [7–9].

Chronic exposure to circulating LCFAs, especially palmitate, contributes to cardiac lipid-induced insulin resistance (CLIR) by promoting intracellular lipid accumulation via the fatty acid transporter CD36. Such myocellular lipid accumulation will disrupt insulin signaling and insulin-stimulated translocation of GLUT4 from the GSV to the plasma membrane. Particularly, the loss of insulin-stimulated glucose uptake is strongly associated with contractile dysfunction [4].

CD36 is normally stored within endosomes and will translocate via transport vesicles to the plasma membrane upon short-term insulin stimulation. Termination of insulin stimulation results in the immediate reinternalization of CD36 within endosomes. In contrast, LCFAs induce a more chronic CD36 translocation to the cell surface, thereby inducing a vicious cycle of lipid-induced lipid accumulation [10]. Accordingly, pharmacological CD36 blocking strategies prevent lipid-induced lipid accumulation in cardiomyocytes [11, 12]. Once accumulated in the cell, lipids trigger the onset of CLIR by inhibiting the insulin signaling pathway, which subsequently leads to reduced insulin-stimulated glucose uptake. At this stage, the heart is considered prediabetic, and cardiomyocytes become mostly dependent on lipid intake for energy provision, which, in the long run, leads to lipotoxicity and contractile dysfunction [10].

CLIR is generally assumed to result from secondary lipid species like ceramide (CER) and diacylglycerol (DAG). Both lipids have been reported to accumulate in cardiomyocytes upon culturing with excess palmitate [13]. CER impairs insulin signaling mainly via inhibiting Akt2 activation [14], and DAG first activates novel PKCs (including PKCθ) prior to inhibition of insulin signaling [15]. However, studies in rodents and humans have found that lipid-induced insulin resistance can also develop without the formation of CER and/or DAG [16–18]. For instance, reduced insulin sensitivity during diabetes progression in humans did not lead to CER or DAG accumulation [16, 17]. Therefore, other mechanisms might be implicated in the onset of CLIR independently of CER and DAG.

One potential contributor is protein S-palmitoylation, a reversible post-translational modification (PTM) where palmitate, upon conversion to palmitoyl-CoA, binds to cysteine residues, thereby regulating protein function and trafficking [19–22]. Interestingly, elevated S-palmitoylation of target proteins has been implicated in metabolic diseases [23, 24]. This modification is mediated by 24 palmitoyl acyltransferases (PATs), also known as DHHC (Aspartate-Histidine-Histidine-Cysteine) enzymes, which exhibit a peculiar intracellular localization and show different expression between tissues [25]. This study investigates the role of S-palmitoylation in CLIR in adult rat ventricular cardiomyocytes (aRVCMs), hypothesizing that excess palmitate elevates protein S-palmitoylation. Given the role of S-palmitoylation in controlling protein function and trafficking [19–22], we hypothesized that basal levels of S-palmitoylation in healthy cardiomyocytes regulate insulin-stimulated GLUT4 translocation and glucose uptake. Next, we assumed that lipid oversupply (i.e., increased palmitate availability) impairs these mechanisms due to elevated protein S-palmitoylation levels, thereby inducing CLIR. Ultimately, identifying specific DHHC enzymes and their protein targets responsible for this modification could lead to novel therapeutic interventions.

Here, we applied pharmacological and genetic intervention strategies, analyzed the cardiomyocyte palmitoylome, and studied the effect of hyper-S-palmitoylation and involvement of specific DHHC isoforms on the induction of CLIR in aRVCMs. Our present findings indicate that S-palmitoylation contributes substantially to the onset of CLIR, in which the S-palmitoylation of the GLUT4 trafficking companion IRAP by DHHC5 is the main event. These findings might offer new avenues for restoring insulin-stimulated glucose uptake and contractile function in lipid-overloaded cardiomyocytes.

## Materials and methods

### Antibodies

The following antibodies were used for Western blotting or acyl-biotinyl exchange analysis: anti-phospho-Akt (pAkt; Ser473; #9271, Cell Signaling Technology), anti-phospho-GSK3β (pGSK3β; Ser9; #9315, Cell Signaling Technology), anti-IRAP (#6918, Cell Signaling Technology), anti-mTORC1 (#2983, Cell Signaling Technology), anti-GAPDH (#2118, Cell Signaling Technology), anti-HA-tag (#3724, Cell signaling Technology), anti-CD36 (MO25, generous gift from Dr. N. Tandon), anti-syntaxin7 (#110072, Synaptic Systems), anti-DHHC2 (#PA5-101994, Thermo Fisher Scientific), anti-DHHC4 (#PA5-113390, Thermo Fisher Scientific), anti-DHHC5 (#21324-1-AP, Thermo Fisher Scientific), anti-SNAP23 (#10825-1-AP, Proteintech), anti-Cav-3 (#610420, BD Transduction Laboratories), anti-PKCθ (Sc-212, Santa Cruz), anti-phospho-PKCθ (#9377, Cell Signaling Technology), and PE-conjugated CD36 (#562702, BD Biosciences). The according IgG-HRP secondary antibodies were used (Cell Signaling Technology).

### Animals

Male Lewis rats of 12 weeks old were purchased from Charles River Laboratories, maintained at the Experimental Animal Facility of Maastricht University in a temperature- and humidity-controlled environment, and subjected to a 12 h light/dark cycle with free access to food and drinking water. All animal experiments were performed according to Dutch regulations and approved by the Dutch Central Committee of Animal Use (CCD) and the Maastricht University Committee for Animal Welfare.

## Isolation and culturing of adult rat cardiomyocytes

### Isolation and culturing of adult rat cardiomyocytes

Rats were anaesthetized intraperitoneally with pentobarbital (200 mg/kg), after which the hearts were rapidly surgically excised. Adult rat ventricular cardiomyocytes were isolated by using a Langendorff perfusion system, as previously described [26]. Atrial and ventricular cardiomyocytes were separated after the Langerhans perfusion step. Briefly, after isolation, ventricular cells were seeded on laminin (#L2020, Sigma-Aldrich) coated plates. After 90 min of adhesion, cells were cultured at 37 °C and 5% CO_2_ in basal low palmitate (LP) medium consisting of M199 medium (#31153, Gibco) supplemented with 5 mM creatine (#C3630, Sigma-Aldrich), 3.2 mM carnitine (#C0283, Sigma-Aldrich), 3.1 mM taurine (#T0625, Sigma-Aldrich), 1% penicillin/streptomycin (P/S; #15140-122, Invitrogen), and 20 µM palmitate (#P0500, Sigma-Aldrich), and palmitate/BSA ratio of 0.3:1. High palmitate (HP) stimulation medium was made of the same composition with the difference in the palmitate concentration (200 µM) and palmitate/BSA ratio of 3:1. To study the effect of S-palmitoylation, cells were exposed to 2-bromopalmitate (2BP; 30 µM, #18263-25-7, Sigma-Aldrich) in LP medium (LP2BP) or HP medium (HP2BP) for the indicated time. To study the effect of DHHC5, cells were exposed to lomitapide (1 µM, #182431-12-5, Simson Pharma Limited) in HP or LP medium for 24 h.

### Culturing of HL-1 cardiomyocytes

HL-1 cells were kindly provided by Dr. W. Claycomb (Louisiana State University, New Orleans, LA, USA) and cultured as previously described [26]. Briefly, HL-1 cells were cultured in gelatin (#48723-500-F, Sigma-Aldrich)/fibronectin (#F1141, Sigma-Aldrich) (G/F) pre-coated flasks at 37 °C and 5% CO_2_ in Claycomb Medium (#51800 C, Sigma-Aldrich) supplemented with 10% fetal bovine serum (FBS; #S181B-500, Biowest), 1% P/S, 0.1 mM norepinephrine (#A7257, Sigma-Aldrich), and 2 mM L-glutamine (#49419, Sigma-Aldrich), which is referred as growth medium. When used for experiments, HL-1 cells were seeded in G/F pre-coated plates in growth medium for 24 h and then changed to control (ctrl) medium, which consists of Dulbecco’s Modified Eagle Medium (DMEM; #31885, Gibco) supplemented with 1% P/S, 0.1 mM norepinephrine, and 1% non-essential amino acids (NEAA; #M7145, Sigma-Aldrich). For the HP condition, ctrl medium was supplemented with 500 µM palmitate, 100 nM insulin (#I5500, Sigma-Aldrich), and a palmitate/BSA ratio of 6:1 for 16 h. To inhibit S-palmitoylation, tunicamycin (3 µM, HPTUN, #T7765, Sigma-Aldrich) or 2BP (30 µM, HP2BP) was added to the HP medium. When studying S-palmitoylation by acyl-biotinyl exchange (ABE, see below), HL-1 cells were treated for 24 h with control medium in addition to 20 µM palmitate to induce basal HL-1 cell S-palmitoylation.

### Transfection of HL-1 cells with siRNA

HL-1 cells were transfected with 10–100 pmol non-coding (n.c) siRNA or siRNA targeting DHHC2, DHHC4, DHHC5, SNAP23, Cav-3, Stx7, and IRAP RNA (all purchased from Thermo Fisher Scientific), using Lipofectamine-RNAiMAX (#13778150, Invitrogen) according to the manufacturer’s instructions. Cells were transfected at a density of 70% confluency for 48 h in transfection medium consisting of Claycomb medium supplemented with 5% FBS and 0.5% L-glutamine before proceeding to experimental treatments as indicated above (see culturing of HL-1 cells). Transfection efficiency was evaluated by Western blot analysis and quantitative PCR.

### Insulin signaling

Insulin signaling was measured as previously described [26]. Briefly, after treating the cells with the different stimulation media, cells were short-term exposed to 100 nM (aRVCMs) or 200 nM (HL-1 cells) insulin for 30 min, followed by lysis in sample buffer and Western blot analysis of pAkt (ser473) and pGSK3β (Ser9).

### Western blot

Equal amounts of cell lysates were subjected to SDS-PAGE and blotted onto activated PVDF membranes. Membranes were blocked in a 5% non-fat dry milk for 1 h at RT, washed with TBS-T, and then incubated overnight at 4 °C with primary antibodies (see antibodies section above). Thereafter, blots were washed, incubated with HRP-linked secondary antibodies, and washed again before visualization. Protein bands were detected using enhanced chemiluminescence (Bio-Rad, Veenendaal, the Netherlands) and quantified by densitometric analysis (Quantity One, Bio-Rad). Data were normalized to Cav-3 or GAPDH protein expression.

### Measurement of glucose uptake

Glucose uptake into HL-1 cardiomyocytes or aRVCMs was measured 24 h after culturing cells in various conditions, after which cells were stimulated with or without 100 nM (aRVCMs) or 200 nM (HL-1 cells) insulin for 30 min at 37 °C. Next, [^3^H]-deoxyglucose uptake was measured by washing the cells three times with uptake buffer (0.46% BSA and 1mM CaCl_2_ in Modified Krebs Ringer (MKR) buffer) and adding a mixture of deoxyglucose (4 µM final) and tracer amounts of [^3^H]-deoxyglucose (0.05 µCi/ml final, #NET328A, Perkin Elmer). After 10 min incubation, the uptake was stopped by two washing steps with ice-cold wash buffer (0.1% BSA, 1 mM CaCl_2_, 0.2 M phloretin (#sc-3548, Santa Cruz), and 0.1% DMSO in MKR buffer). Cells were lysed in 1 M NaOH and radioactivity was measured via liquid scintillation counting with a β-counter (Tri-Carb 2910 TR, Perkin Elmer). Data were normalized to total protein content, as determined by Bradford assay.

### Measurement of cardiomyocyte contractile dynamics

**Fig. 1:**
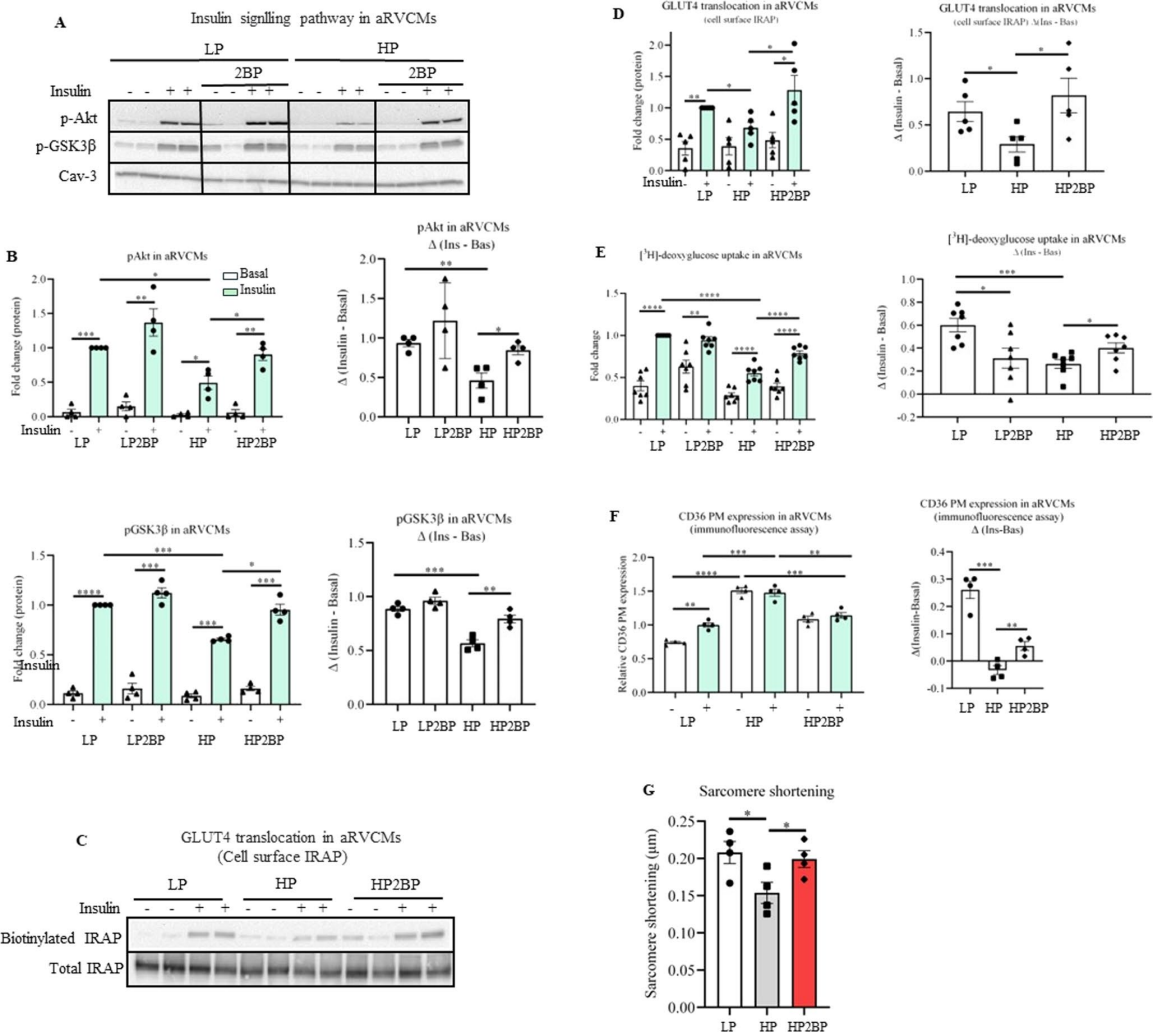
S-palmitoylation impairs insulin sensitivity, substrate transporter trafficking (GLUT4 and CD36), insulin-stimulated glucose uptake and contractile function in high palmitate-treated cardiomyocytes. Adult rat ventricular cardiomyocytes (aRVCMs) were cultured for 24 h in low (LP) or high palmitate (HP) medium, treated without or with 30 µM 2-bromopalmitate (2BP) supplementation (LP2BP and HP2BP). Thereafter, cells were treated for 30 min without (-, white bars) or with insulin (+, light green bars, 100 nM). Insulin sensitivity, cell surface GLUT4 translocation, CD36 plasma membrane (PM) expression, glucose uptake, and sarcomere shortening were assessed. **A**, **B**) Representative blot (**A**) and quantification of pAkt (ser473) and pGSK3β (Ser9) (**B**) protein expression in aRVCMs treated with different conditions (*n* = 4) as measurement for insulin sensitivity. Caveolin-3 (Cav-3) was used as loading control. **C**-**D**) Representative blot (**C**) and quantification (**D**) of GLUT4 translocation and total lysate fractions in aRVCMs treated with different conditions (*n* = 5). (**E**) Quantification of [^3^H]-deoxyglucose uptake in aRVCMs treated upon different conditions (*n* = 8). (**F**) Quantification of the CD36 plasma membrane (PM) expression in aRVCMs was assessed with fluorescent-labelled CD36 antibody (*n* = 4). In panel B, D, E, and F the insulin effect (Δinsulin; right graph) is displayed for each condition, representing the calculated difference (Δ) between - and + insulin stimulation. (**G**) Contractile function was assessed based on sarcomere shortening (*n* = 4; Imaging of 10 cells measurement/condition). Data expressed as means ± SEM *p < 0.05, **p < 0.01, ***p < 0.001, ****p < 0.0001 by two-tailed unpaired Student’s t-test

Cardiomyocyte contractile dynamics were measured in aRVCMs 24 h after culturing the cells with different stimulation media. To investigate the parameters in Fig. 1 and supplemental Figure S7, Contractile properties of aRVCMs were then measured using a video-based cell geometry system (IonOptix, Milton). Cells were electrically paced in Tyrode buffer at 20 V, 1 Hz, and 37 °C. Line-scan images of 10–15 successive beats were recorded with IonWizard acquisition software for the calculation of contractile parameters; sarcomere shortening, RT50, and time to peak. To investigate cell shortening in Fig. 7 and supplemental Figure S16, contractile properties of aRVCMs were analyzed under 30 V and 1 Hz electric field stimulation. Recordings of contracting cells were made by bright-field microscopy and analyzed with FIJI (version 1.54). In more detail, a 6-well plate containing the cells with electrodes coupled on top was placed on a heating stage (Tokai Hit) set at 39 °C on top of the microscope stage of an inverted microscope (CK2, Olympus). A camera (MikroCamII, Bresser GmbH) recorded the contraction videos with 30 fps at 1824 × 1216 pixels with a 10X objective. The field of view was 1404 μm with 3–8 cells visible in each video. Cells that were overlapping, not contracting, exhibiting apoptosis, or not fully in view were not analyzed. Videos were imported into FIJI and segmented using the intensity threshold method (default algorithm). Segmented images were subsequently processed with the Analyze particles function of FIJI to measure the area, minor, and major axes of the cells in each frame. The Beat Analysis tool (https://martijnhoes.shinyapps.io/myomate) was used to calculate the cell shortening.

### Surface-protein biotinylation for detecting GLUT4 and CD36 translocation

Surface-protein biotinylation was measured as previously described [26]. Briefly, after a 24 h culture in various conditions, aRVCMs were incubated for 30 min with or without 100 nM insulin. During the last 10 min of this period, the cell-impermeable reagent sulfo-NHS-LC-biotin (#21335, Thermo Fisher Scientific) was added. Thereafter, the cells were washed and lysed for subsequent IP with streptavidin-agarose beads (#20359, Thermo Fisher Scientific). After further washing and elution of the biotinylated proteins from the beads, samples containing the biotinylated proteins were used for Western blot analysis of CD36 and insulin-regulated aminopeptidase (IRAP, which reflects GLUT4 trafficking).

### CD36 plasma membrane expression

CD36 plasma membrane expression was measured 24 h after culturing cells in various conditions or upon viral transduction, after which cells were stimulated with or without 100 nM insulin for 30 min at 37 °C. Next, cells were incubated with a PE-conjugated CD36 antibody (#562702, BD Biosciences) for 45 min at 4 °C in PBS (1:400). Fluorescence was measured at an excitation wavelength of 560 nm and an emission wavelength of 578 nm using the SpectraMax iD3 Multi-Mode Microplate Reader (Molecular Devices).

### RNA extraction and real-time quantitative PCR

Total RNA from the cells was isolated using QIAzol (#79306, Qiagen) and the RNeasy mini kit (#74106, Qiagen), according to the manufacturer’s guidelines. Complementary DNA (cDNA) was synthesized using qScript™ cDNA SuperMix (#95048-500, Quantabio) according to the manufacturer’s instructions. Real-time quantitative PCR was conducted on a Step One Plus detection system (Applied Biosystems). Cycle conditions were 95 °C for 20 s, followed by 40 cycles of 95 °C for 3 s, and 60 °C for 30 s. The PCR reaction mixture contained SYBR green master mix (#A25742, Thermo Fisher Scientific), 0.3 µM forward and reverse primer (IDT technologies), RNase-free water, and 12.5 ng cDNA template. Data were analyzed using the comparative ΔΔCt method and normalized to the most stable reference genes. Primer sequences are available upon request.

### Acyl-biotinyl exchange (ABE) assay for detecting S-palmitoylation of specific proteins

The acyl-biotinyl exchange (ABE) assay was performed as described by Wan et al., [27]. Cells were lysed in a buffer containing 150 mM NaCl, 50 mM Tris, 5 mM EDTA, pH 7.4, 0.1% SDS, and 1.7% Triton X-100, with 1 × protease inhibitor cocktail (#04693116001, Sigma-Aldrich) and 10 mM NEM (#23030, Thermo Fisher Scientific), which protects free cysteines from capture by thiol-reactive resin. After three chloroform-methanol precipitations, pellets were resuspended in a buffer containing 8% SDS, and the sample was divided into two equal fractions. One fraction was incubated with 0.7 M hydroxylamine (+ HA; #8.14441, Sigma-Aldrich), a thioester cleavage agent for S-S-palmitoylation sites, while the rest of the proteins were incubated with Tris (-HA). The omission of hydroxylamine serves as a negative control. Since the thioester bonds at S-S-palmitoylation sites in -HA fraction were not cleaved, proteins captured by the thiol-reactive resin represented non-specific binding. Samples were rotated for 1 h at room temperature. After a chloroform-methanol precipitation, the pellets were resuspended in a buffer containing EZ-Link HPDP-Biotin (#21341, Thermo Fisher Scientific) and incubated for 1 h at room temperature. Unreacted HPDP-biotin was removed by chloroform-methanol precipitation and pellets were resuspended in lysis buffer. Samples were diluted to 0.1% SDS and biotinylated proteins were affinity-purified using streptavidin-agarose beads (#20359, Thermo Fisher Scientific). 1% Beta-mercaptoethanol (#BP176, Fisher Scientific) was used to cleave HPDP-biotin and release biotinylated proteins from the beads. Finally, proteins were denatured in sample buffer. One fraction of the eluate was sent to Maastricht Proteomics Center for analysis with Liquid Chromatography-tandem mass spectrometry (LC-MS-MS) analysis. The other fraction was analyzed using SDS-PAGE/Western blot or SDS-PAGE/silver staining to confirm specific protein S-palmitoylation (Western blot) or general protein S-palmitoylation (silver staining).

### Rat model of hyperlipidemia

Hyperlipidemia was induced as previously described, generating an early-stage model of diabetes disease presenting with mild hyperglycemia and hyperinsulinemia [11, 28]. Briefly, male Wistar rats (starting body weight ≈ 300 g; Envigo) were fed a high-fat diet (cat. no. 829197, 60% calories from fats; Special Diet Services) for 5 weeks, and on day 14, they received a single low-dose intraperitoneal injection of streptozotocin (25-mg/kg body weight). Control rats were fed a standard chow diet for 5 weeks. Experiments conformed to the Home Office Guidance on the Operation of the Animals (Scientific Procedures) Act, 1986, and were approved by a local ethics committee (University of Oxford, United Kingdom) [29].

### Acyl-resin assisted capture for in vivo IRAP-hyper-S-palmitoylation detection

S-acylated proteins were purified from snap-frozen tissue using acyl resin-assisted capture [30]. 10 mg of tissue was incubated in blocking buffer (2.5% SDS (w/v), 100-mmol/L HEPES, 1-mmol/L EDTA and pH 7.4) and free cysteines alkylated by addition of 100-mmol/L N-ethylmaleimide and incubated at 40 °C for 4 h. Excess maleimide was removed by acetone precipitation; protein pellets were washed with 70% acetone (v/v), dried, and resolubilized in binding buffer (1% SDS [w/v], 100-mmol/L HEPES, 1-mmol/L EDTA, and pH 7.4). S-acylated proteins were captured on thiopropyl sepharose resin in the presence of 200-mmol/L hydroxylamine (pH 7.4) for 2.5 h at room temperature. An identical reaction in which hydroxylamine was replaced with 200-mmol/L NaCl served as a negative control. Following the capture of acylated proteins, the beads were extensively washed in binding buffer, and proteins were eluted by heating for 10 min at 60 °C in SDS-PAGE (polyacrylamide gel electrophoresis) loading buffer supplemented with 100-mmol/L DTT. The input fraction, negative control (− HA [minus hydroxylamine]), and S-acylated (+ HA) fractions were analyzed to assess the enrichment of the S-acylated fraction by dividing the + HA fractions by the input fraction.

### *In vitro* radiometabolic labeling

To concentrate the palmitate label from the ^14^C-palmitate ethanol stock solutions, ^14^C-palmitate was dried under vacuum and redissolved in MKR buffer 1X + 0,1% BSA with α-cyclodextrin 10 mg/ml (#C4642, Sigma-Aldrich) before adding to cell suspension for 1 h with or without 2BP. Cells were lysed, and an equal amount of protein was loaded onto the gel for silver staining.

### Silver staining of gels

After SDS-PAGE, gels were fixed in 30% ethanol and 10% acetic acid (#695092, Sigma-Aldrich) for 60 min. Subsequently, fixation bath was renewed and left overnight. The day after, the gel was sensitized using a tetrathionate sensitizing solution and, after rinsing with water, protein bands were detected by impregnating the gel with silver nitrate. Stop solution was added when the protein bands were well visible on the gel.

### Mass Spectrometry procedure

1. In-liquid digestion

5–20 µg total protein was dissolved in 25 µl 50 mM ammonium bicarbonate (ABC; #A6141, Sigma-Aldrich) with 5 M urea (#51458, Fluka). 2.5 µL of TCEP solution (200 mM final; # 52486, Sigma-Aldrich) was added and incubated at 45 °C for 45 min. The proteins were alkylated by adding 3 µL of iodoacetic acid solution (40 mM final; #35603, Thermo Fisher Scientific). The reaction took place at room temperature for 45 min in the dark. For the digestion, 1 µg trypsin/lysC was added to the protein and incubated at 37 °C for 2 h. 100 µl of 50 mM ABC was added to dilute the urea concentration and further incubated at 37 °C for 18 h. The digestion mix was centrifuged at 2,500 g for 5 min, and the supernatant was collected for LCMS analysis.

2) Protein identification using LC-MS/MS

A nanoflow HPLC instrument (Dionex ultimate 3000) was coupled on-line to a Q Exactive (Thermo Scientific) with a nano-electrospray Flex ion source (Proxeon). 5 µl of the digest was loaded onto a C18-reversed phase column (Thermo Scientific, Acclaim PepMap C18 column, 75 μm inner diameter x 50 cm, 2-µm particle size). The peptides were separated with a 240 min linear gradient of 4–68% buffer B (80% acetonitrile and 0.08% formic acid) at a flow rate of 300 nl/min. MS data was acquired using a data-dependent top-10 method, dynamically choosing the most abundant precursor ions from the survey scan (280–1400 m/z) in positive mode. Survey scans were acquired at a resolution of 70,000 and a maximum injection time of 120 ms. Dynamic exclusion duration was 30 s. Isolation of precursors was performed with a 1.8 m/z window and a maximum injection time of 200 ms. Resolution for HCD spectra was set to 30,000 and the Normalized Collision Energy was 32 eV. The under-fill ratio was defined as 1.0%. The instrument was run with peptide recognition mode enabled, but exclusion of singly charged and charge states of more than five.

3 Database search and quantification

The MS data were searched using Proteome Discoverer 2.2 Sequest HT search engine (Thermo Scientific), against the UniProt rat database. The false discovery rate was set to 0.01 for proteins and peptides, which had to have a minimum length of 6 amino acids. The precursor mass tolerance was set at 10 ppm and the fragment tolerance at 0.02 Da. One miss-cleavage was tolerated. NEM modification (+ 125.048) and carbamidomethylation of cysteines as well as oxidation of methionine were set as a dynamic modification. Label free quantitation was conducted using the Minora Feature Detector node in the processing step and the Feature Mapper node combined with the Precursor Ions Quantifier node in the consensus step with default settings within Proteome Discoverer 2.2.

### Adenovirus construction, amplification, and transduction of aRVCMs

A total of seven Adenoviral constructs were constructed and purchased from VectorBuilder GmbH (Neu-Isenberg, Germany): 1 control adenovirus containing enhanced green fluorescent protein (eGFP) (Ad GFP), 3 recombinant adenoviruses encoding full-length wild-type (WT) human Stx7, Cav-3, and IRAP (WT constructs), and 3 recombinant adenoviruses containing the S-palmitoylation-deficient version of Stx7, Cav-3, and IRAP (ΔCys constructs). Each construct bears eGFP followed by HA-tag genes at the C-terminus of every protein in order to distinguish the transduced proteins from the endogenous ones. For each protein, the mutations are explained below.

- **Cav-3**: A total of 5 cysteine residues were mutated in Cav-3: Cys19, 106, and 116 were replaced by alanine, while Cys129 and 140 were mutated to serine to minimize the nucleotide exchange in the Cav-3 DNA sequence (Cys◊Ala; Cys◊Ser = ΔCys-Cav-3). Swiss Palm predicts 4 palmitoylated cysteine residues in Cav-3 (19, 106, 116, and 129 https://swisspalm.org/proteins/P51638), while we identified an additional cysteine residue (Cys140) in our palmitoylome analysis (Table S2). Our finding is also supported by Dr. M.S. Bhogal, who identified S-palmitoylation of Cys140 (besides Cys 19, 106, 116, and 129 residues in Cav-3) (https://badrilla.com/2017/06/05/acyl-peg-exchange-an-important-advance-in-the-S-palmitoylation-toolbox/).
- **Stx7**: Cysteine 28 and 239 were mutated into alanine in Stx7 (Cys◊Ala = ΔCys-Stx7). While Cys239 is predicted to be palmitoylated by SwissPalm (https://swisspalm.org/proteins/O70257), in agreement with the literature [31], we also identified S-acylation at Cys28 thanks to our LC-MS/MS analysis (Table S2).
- **IRAP**: Cysteine residues 103 and 114 were mutated into alanine in IRAP (Cys◊Ala = ΔCys-IRAP). The two cysteines were found to be palmitoylated in transfected HEK293T cells and 3T3-L1 adipocytes [32, 33]. Additionally, we confirmed IRAP Cys103 S-palmitoylation through LC-MS/MS analysis of the cardiac palmitoylome (Table S2).

Each plasmid construct was amplified in Luria-Bertani medium supplemented with Ampicillin 100 mg/ml for positive selection. After isolation of plasmid DNA with Midi-Prep (Invitrogen Kit), each adenoviral construct was linearized through *PacI* enzymatic digestion (*PacI* restriction site within the constructs). Constructs were transfected in HEK293T cells for the creation and amplification of the viral particles. After 3 rounds of amplification in HEK293T cells, we isolated the viral particles that were transduced in aRVCMs for functional assays. aRVCMs were transduced with a multiplicity of infection (MOI) of 50 for 24 h after which the medium was replaced by the different stimulation media.

### Statistical analysis

Data were statistically analyzed using GraphPad Prism v5 and are reported as mean ± SEM. The D’Agostino and Pearson omnibus normality test was used to test for normal distribution. When datasets were normally distributed, a two-tailed unpaired Student’s t‐test (with Welch’s correction if necessary) was used to determine statistical significance between groups. If datasets did not pass normality, the Kruskal‐Wallis or Mann‐Whitney analysis was applied. p values < 0.05 were considered to indicate a significant difference (*, *p* < 0.05; **, *p* < 0.01; ***, *p* < 0.001; ****, *p* < 0.0001).

## Results

### S-palmitoylation impairs insulin sensitivity, substrate transporter trafficking, insulin-stimulated glucose uptake and contractile function in high palmitate-treated cardiomyocytes

Prior to testing the role of S-palmitoylation in our in vitro cardiac cell models of insulin resistance, we investigated whether in adult rat ventricular cardiomyocytes (aRVCMs) exposed to high palmitate (HP), accumulated lipids could directly induce maladaptive signaling actions, thereby inducing CLIR. For this, we focused on DAG-mediated activation of PKC-θ, which can be assessed by increased phosphorylation at Thr538 [34]. This phosphorylation event appeared not to be altered by HP (Fig. S1). Hence, this result sets the stage for a possible important contribution of S-palmitoylation in CLIR.

A useful tool in studies on the role of S-palmitoylation in cellular function is 2-bromopalmitate (2BP), the most widely accepted and commonly used S-palmitoylation pan-inhibitor. 2BP directly inhibits DHHCs by covalently modifying their active site [35]. By studying the incorporation of radiolabeled palmitate into protein, we confirmed that at an optimal concentration of 30 µM 2BP inhibits protein S-palmitoylation in aRVCMs (Fig. S2A). Moreover, 2BP prevented the increase in protein S-palmitoylation induced by HP exposure in aRVCMs (Fig. S2B).

Hereafter, we tested the impact of S-palmitoylation on the onset of CLIR in our in vitro models of insulin resistance. First, we assessed the effect of 2BP on the insulin signaling pathway in ex vivo isolated aRVCMs. For evaluation of insulin signaling, phosphorylation levels of Akt2 (pAkt Ser473) and its downstream kinase glycogen synthase kinase-3β (pGSK3β Ser9) were analyzed. HP supplementation induced a significant decrease in insulin-stimulated phosphorylation of Akt and GSK3β compared to low palmitate (LP) supplementation (Fig. 1A-B), in agreement with our previous studies [10, 36]. Treatment with 2BP preserved Akt and GSK3β phosphorylation, and hence insulin signaling, in lipid-overloaded aRVCMs (Fig. 1A-B). Additionally, we treated cardiomyocytes with another commonly used inhibitor of S-palmitoylation, tunicamycin, which reduces S-palmitoylation by competing for binding of the lipid donor palmitoyl-CoA to the DHHCs [37]. Similar to 2BP, tunicamycin was observed to preserve insulin signaling in aRVCMs (Fig. S3).

After confirmation that GLUT4 expression exceeds that of GLUT1 in aRVCMs (Fig. S4), we assessed the effect of S-palmitoylation on GLUT4 translocation by measuring the cell surface content of the GLUT4 companion insulin-responsive aminopeptidase (IRAP) in aRVCMs. As previously reported in our studies [26, 36], HP supplementation inhibited insulin-stimulated IRAP plasma membrane translocation, which was reversed by co-incubation with 2BP. Hence, inhibition of S-palmitoylation not only preserved insulin signaling upon HP supplementation but also prevented the loss of insulin-stimulated GLUT4 translocation (Fig. 1C-D). Concomitantly, we used a radiolabeled glucose analog ([^3^H]-deoxyglucose) to measure myocellular glucose uptake. As expected [10, 26], insulin-stimulated glucose uptake was significantly decreased upon HP exposure compared to LP culturing (Fig. 1E). Co-treatment with 2BP partially preserved insulin-stimulated glucose uptake (Fig. 1E). These findings were also confirmed in HL-1 cardiomyocytes (Fig. S5).

Finally, trafficking of the fatty acid transporter CD36 was investigated using two methods: immunofluorescence measurement and cell surface biotinylation assay. In line with our previous results [26], we confirmed that HP induced an increase in cell surface CD36 relative to LP. Different from the LP condition, insulin did not further increase CD36 translocation to the plasma membrane in the HP condition (Fig. 1F, Fig. S6). 2BP treatment significantly reversed elevated CD36 translocation (Fig. 1F, Fig. S6) and restored insulin-stimulated CD36 translocation in HP-exposed aRVCMs (Fig. 1F). Next, we measured the impact of S-palmitoylation on the contractile function of aRVCMs. Sustained intracellular accumulation of palmitate is known to induce cardiac contractile dysfunction [10]. Accordingly, in the present study, HP exposure resulted in a ~ 25% decrease in sarcomere length. (Fig. 1G; for other contraction parameters see Fig. S7). This pattern of contractile changes is in close agreement with our previous work [26, 36], 2BP treatment prevented HP-induced decrease in sarcomere shortening (Fig. 1G). Hence, inhibition of general protein S-palmitoylation preserves contractile function in lipid-overexposed cardiomyocytes.

Altogether, these results suggest that CLIR prevention may be achieved by inhibiting S-palmitoylation.

### Transient DHHC5 knockdown partially preserves insulin-stimulated glucose uptake in lipid-overexposed HL-1 cardiomyocytes

Since 2BP effectively inhibits all DHHC isoforms, we next aimed to unravel the involvement of specific DHHC isoforms in mediating the aberrant metabolic changes in lipid-overloaded cardiomyocytes. Each of the 24 existing DHHCs is detectable in the rat heart at the mRNA level. However, half of the DHHCs display low, if barely any, mRNA expression [38]. There are 11 DHHCs detected at the protein level in the heart [39]. In view of their localization in the plasma membrane and in endosomes, i.e., the sites of GLUT4 translocation machinery, we selected DHHC2, DHHC4, and DHHC5 for transient knockdown through siRNA silencing in cardiomyocytes. These transient gene silencing experiments were performed in HL-1 cells because, unlike aRVCMs, HL-1 cells can take up siRNA/lipofectamine complexes [26].

**Fig. 2:**
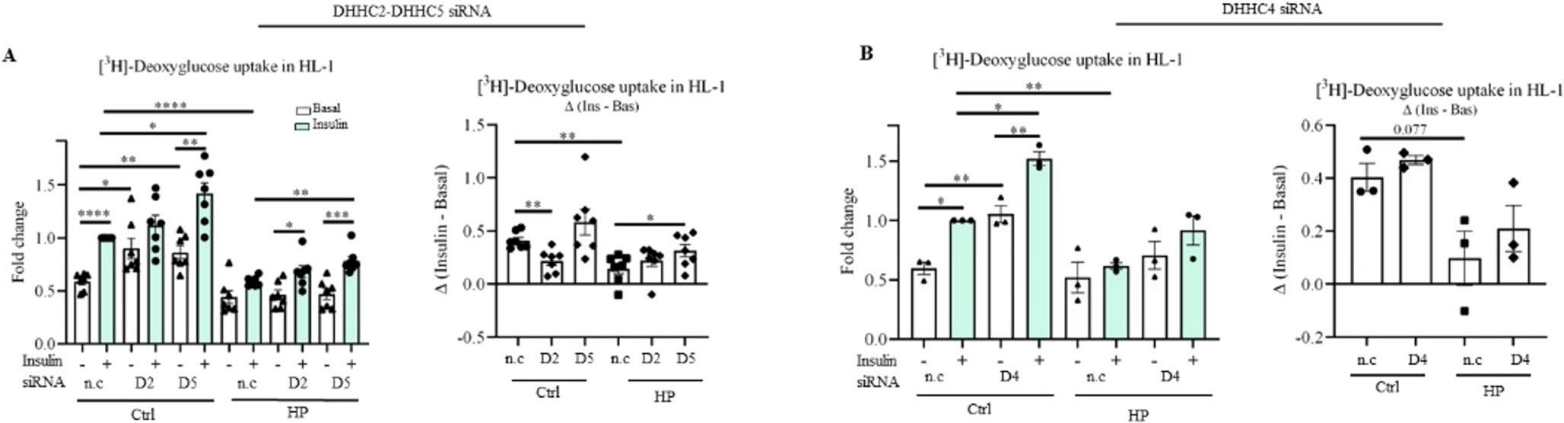
Transient DHHC5 enzyme knockdown partially preserves insulin-stimulated glucose uptake in lipid-overexposed HL-1 cardiomyocytes. HL-1 cells were silenced with siRNAs against non-coding (n.c), DHHC2 (D2), DHHC4 (D4), and DHHC5 (D5) for 48 h in transfection medium, then stimulated for 16 h with control (Ctrl) or high palmitate (HP) medium, and subsequently incubated for 30 min without (-, white bars) or with insulin (+, light green bars, 200 nM). Glucose uptake was assessed. **A**-**B**) Quantification of [^3^H]-deoxyglucose uptake in HL-1 cells after treatment with non-coding (n.c), D2, and D5 siRNA (*n* = 7) (A) or after treatment with D4 siRNA (*n* = 3) (B). [^3^H]-deoxyglucose uptake values are displayed as fold changes normalized from the ‘n.c. + insulin’ condition’ (left panel), after which the Δinsulin was calculated (right panel). Data expressed as means ± SEM *p < 0.05, **p < 0.01, ***p < 0.001, ****p < 0.0001 by two-tailed unpaired Student’s t-test

First, 48 h after transfection using siRNAs complexed with lipofectamine, transient knockdown of the DHHCs of interest was assessed both at protein and mRNA levels. Transfection with DHHC2 and DHHC5 siRNAs decreased protein expression of these DHHC isoforms in control and in HP-exposed HL-1 cardiomyocytes, respectively (Fig. S8A-B). We were unable to verify the downregulation of protein expression of DHHC4 because of a lack of signal from the anti-DHHC4 antibody in the Western blot (data not shown). However, mRNA expression levels of *Dhhc2*, *Dhhc4*, and *Dhhc5* were greatly decreased compared to control-transfected cells (Fig. S8C). We then measured [^3^H]-deoxyglucose uptake and observed more than 50% decrease in insulin-stimulated glucose uptake in HP-exposed HL-1 cells compared to control (Fig. 2A-B). Treatment with DHHC5 siRNA, but not with DHHC2 or DHHC4, significantly increased insulin-stimulated glucose uptake (i.e., by 40%) during HP exposure in HL-1 cells (Fig. 2A-B). Additionally, in the control condition, DHHC4 and DHHC5 knockdown resulted in a significant increase in both basal and insulin-stimulated glucose uptake (Fig. 2A-B). Collectively, these data suggest that DHHC5-mediated protein S-palmitoylation in HP-exposed cardiomyocytes is at least one of the mechanisms limiting insulin-stimulated glucose. Besides its maladaptive effect on glucose uptake, DHHC5 also appears to mediate the increased translocation of CD36 in HP-exposed cardiomyocytes. Similarly to 2BP, the specific DHHC5 inhibitor lomitapide [40] reversed HP-induced elevated CD36 translocation (Fig. S9), thereby exposing a vicious cycle between increased CD36-mediated uptake of palmitate (i.e., DHHC5’s substrate) and DHHC5 induction of CD36 translocation.

### Analysis of the rat cardiac S-palmitoylome by LC-MS/MS

Having established that targeting protein S-palmitoylation can restore HP-induced alterations in glucose uptake, we aimed to identify the S-palmitoylated proteins in cardiomyocytes and to select the ones with possible involvement in GLUT4 translocation. We hypothesized that S-palmitoylation of the component(s) of the glucose uptake machinery would be altered in HP-lipid overload conditions compared to LP-basal state, thereby impairing insulin-stimulated glucose uptake in cardiomyocytes. Thus, we began by analyzing the cardiac S-palmitoylome in isolated aRVCMs cultured for 24 h in LP-basal medium to profile physiologically relevant S-palmitoylated proteins. We chose to investigate the cardiac S-palmitoylome in this basal condition to reduce artifactual enrichment of proteins that associate nonspecifically with membranes when extracellular palmitate is elevated, which can complicate MS identification.

**Fig. 3:**
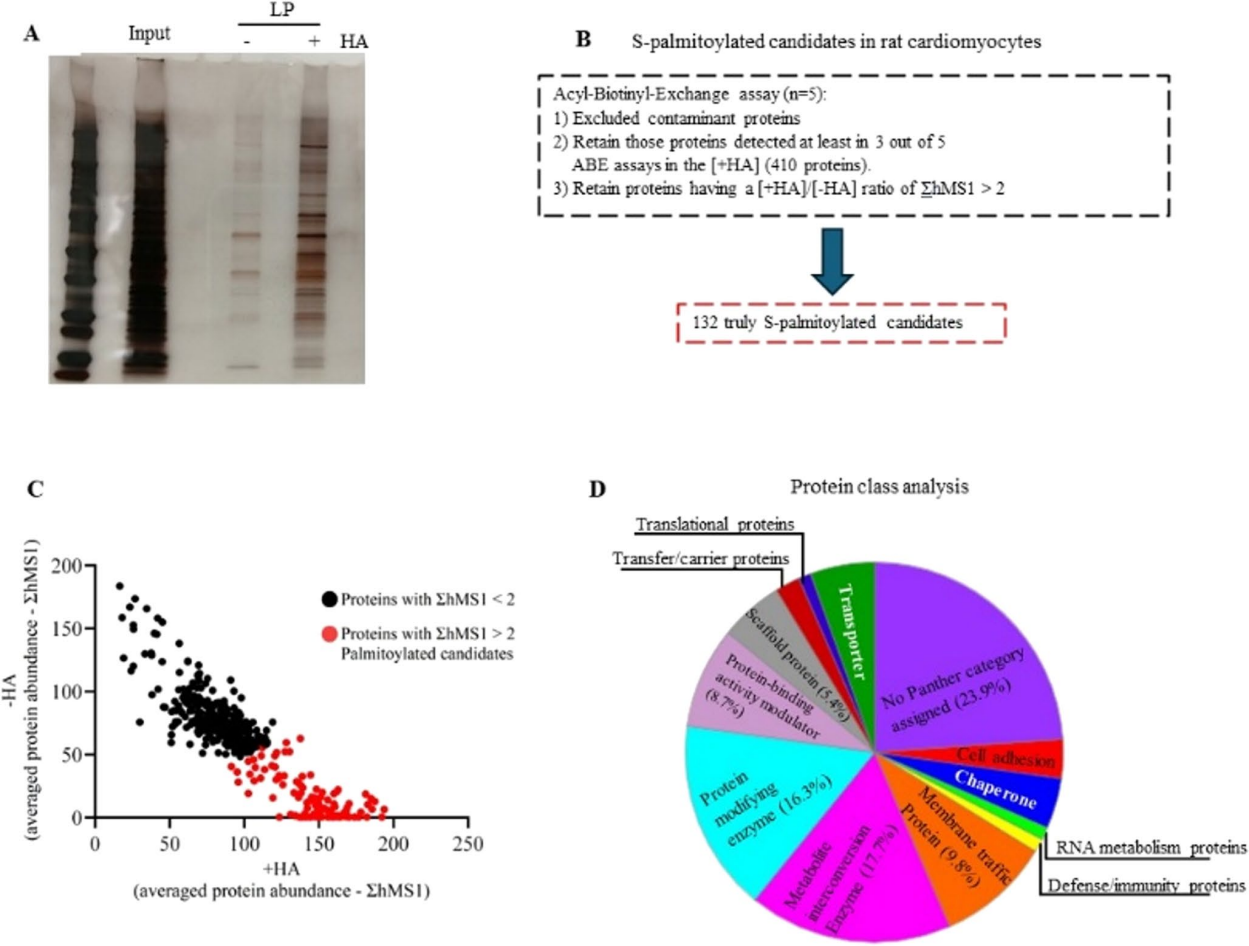
Evaluation of the quality of the acyl-biotin-exchange (ABE) and analysis of the cardiac S-palmitoylome

LP cultured aRVCMs were then subjected to the acyl-biotinyl-exchange (ABE) assay to enrich S-palmitoylated proteins [27]. Gel electrophoresis of the ABE-immunoprecipitate followed by silver staining confirmed that the hydroxylamine (HA) treatment (+ HA fraction) step in the ABE procedure was effective in enriching for palmitoylated proteins compared to the fraction of the sample where HA treatment was omitted (-HA fraction) (Fig. 3A). The two fractions [-HA] and [+ HA] were analyzed by LC-MS/MS.

After excluding contaminant proteins, we retained only those present in at least 3 out of 5 experiments in the [+ HA] fraction, obtaining a list of 410 hits. In this list, only 132 are considered truly S-palmitoylation candidates (Fig. 3B, Table S1), as they are enriched at least 2-fold in the [+ HA] fraction (depicted as red dots in Fig. 3C).

The 132 candidate S-palmitoylated proteins were analyzed using the Panther Classification System to determine their associated protein classes (Fig. 3D). To identify proteins with potential relevance to GLUT4 translocation and cardiac glucose uptake, each class was examined through manual curation using UniProt entries and current literature. Protein classes representing less than 5% of the total dataset, including “transporters”, “translation proteins”, “transfer/carrier proteins”, “RNA-metabolism proteins”, “defense/immunity proteins”, “chaperones”, and “cell-adhesion proteins”, were analyzed first and excluded from further consideration, as none of their members showed evidence of involvement in GLUT4 translocation. The two largest remaining categories, “no classification” (23.9%) and “metabolite interconversion enzymes” (17.7%), also did not contain possible proteins involved in GLUT4 translocation.

From the remaining categories, four candidates were selected for further investigation (Table S2). Two candidates (SNAP23 and Syntaxin-7) belonged to the category of “membrane trafficking proteins”, one candidate (Caveolin-3) fell into the “scaffold proteins”, and the last one (insulin-regulated aminopeptidase; IRAP) into the “protein-modifying enzymes” (Table S2). Based on our curation, we summarize below the current knowledge regarding the roles of these four proteins in GLUT4 translocation and glucose uptake.

#### SNAP23

SNAP23 belongs to the t-SNARE proteins, a known membrane trafficking component that mediates tethering and fusion of vesicles with target membranes [41, 42]. For instance, SNAP23 is essential for the docking and merging of GLUT4 storage vesicles (GSVs) with the plasma membrane in adipocytes [43]. Moreover, overexpression of a truncated version of SNAP23 or SNAP23 knockdown inhibited insulin-stimulated glucose uptake in adipocytes [44]. Interestingly, S-palmitoylation promotes SNAP23 membrane fusion in mast cells and also between synthetic vesicles in cell-free systems [45, 46].

#### Syntaxin-7

Syntaxin-7 (Stx7) is another member of the t-SNARE protein superfamily. In several mammalian cell lines, Stx7 was found to be localized to endosomes and suggested to be involved in protein trafficking between the plasma membrane and endosomes, as well as in vesicle fusion at the endosomes [31, 47], However, it is not known whether Stx7 contributes to the regulation of glucose uptake. As a side note, syntaxin-8 was also among the palmitoylated proteins in the heart (Table S1), but it was not selected for the shortlist because it is reported to promote retrograde transport from cis-Golgi membranes to the endoplasmic reticulum (ER) [47], and, therefore, is unlikely to be involved in glucose uptake nor in endosome-plasma membrane trafficking.

#### Caveolin-3

Caveolin-3 (Cav-3) is a so-called ‘scaffold protein’ and is responsible for the organization of caveolar rafts within the plasma membrane. Insulin signaling proteins are present within caveolae, most notably the insulin receptor (IR). Furthermore, Cav-3 knockdown abolished insulin-stimulated glucose uptake in the HL-1 cell line [48].

#### IRAP

Insulin-responsive aminopeptidase (IRAP) is classified a “protein-modifying enzyme” due to its known function to degrade peptides such as oxytocin and vasopressin [49]. However, IRAP is also the companion of GLUT4, and it is abundantly expressed in the GSVs, a specific intracellular non-endosomal network of small vesicles dedicated to GLUT4 translocation in response to different stimuli, most notably insulin [9, 50]. Together, IRAP and GLUT4 traffic to the plasma membrane in response to insulin [26, 51]. Moreover, IRAP deficiency was reported to impair insulin-stimulated glucose uptake in adipocytes and skeletal muscle [52]. However, the function of IRAP S-palmitoylation is still unknown.

In subsequent steps, each of these proteins (Table S2) was further investigated to confirm its role in cardiac glucose uptake.

Adult rat ventricular cardiomyocytes (aRVCMs) were cultured for 24 h in low palmitate (LP) medium. Upon cell lysis, an ABE assay was performed. **(A)** Representative silver staining of the ABE assay to assess the quality of the assay in enriching S-palmitoylated proteins. **(B)** Flowchart of data analysis and selection criteria. **(C)** Each of the 410 proteins found in [+ HA] (hydroxylamine) fractions upon LC-MS/MS in at least 3 out of 5 ABE assays is plotted as averaged + HA ΣhMS1 (ΣhMS1 = the sum of the height of the peaks of all the detected unique peptides for a given protein) against averaged -HA ΣhMS1. Black dots represent proteins having a ratio of ΣhMS1 < 2, while red dots are proteins (132) having a ratio of ΣhMS1 > 2 and are considered truly S-palmitoylated candidates since they are enriched more than 2-fold in the + HA fraction. **(D)** The 132 truly S-palmitoylated candidates were submitted to Panther System Analysis for protein classification. Pie chart of protein classification. A total of 132 palmitoylated candidates are matched to 13 protein classes. From there, we selected four final S-palmitoylated candidates for further investigation (Table S2).

#### SNAP23, Cav-3, Stx7, and IRAP contribute to the regulation of glucose uptake in rodent cardiomyocytes

**Fig. 4:**
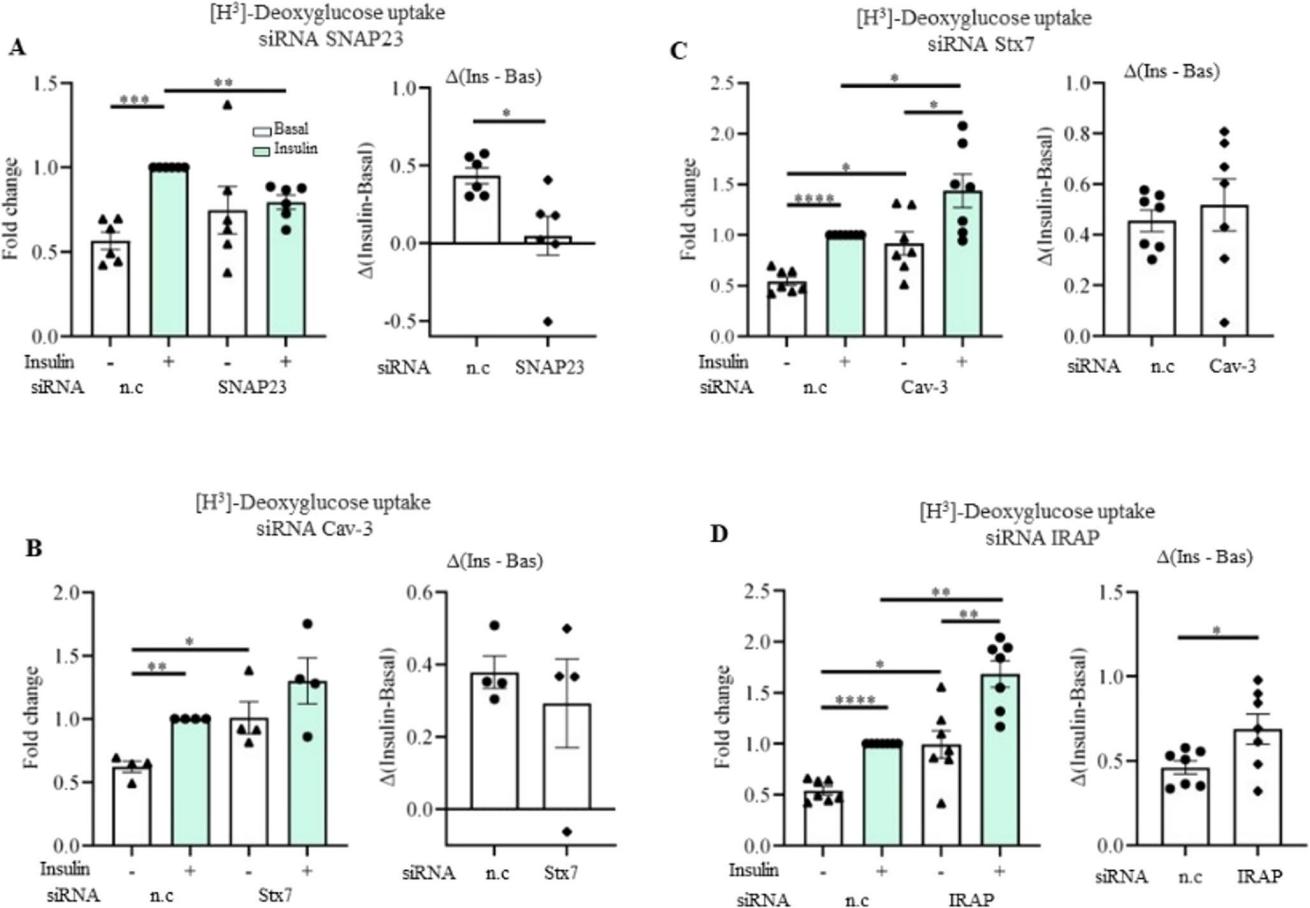
Glucose uptake in HL-1 cardiomyocytes upon transient knockdown of SNAP23, Stx7, Cav-3, and IRAP. HL-1 cells were silenced with siRNAs against non-coding (n.c), SNAP23, caveolin-3 (Cav-3), syntaxin-7 (Stx7), and IRAP for 48 h in transfection medium, then stimulated with control (Ctrl) medium for 16 h, and subsequently incubated for 30 min without (-, white bars) or with insulin (+, light green bars, 200 nM). Glucose uptake was assessed. **A**-**D**) Quantification of [^3^H]-deoxyglucose uptake in HL-1 cells after treatment with n.c and SNAP 23 siRNA (*n* = 6) (**A**), Cav-3 siRNA (*n* = 4) (**B)**, Stx7 siRNA (*n* = 7) (**C**), and IRAP siRNA (*n* = 7) (**D**). [3H]-deoxyglucose uptake values are displayed as fold changes normalized from the ‘n.c. + insulin’ condition’ (left panel), after which the Δinsulin was calculated (right panel). Data expressed as means ± SEM *p < 0.05, **p < 0.01, ***p < 0.001, ****p < 0.0001 by two-tailed unpaired Student’s t-test

To study the putative role of SNAP23, Cav-3, Stx7, and IRAP in cardiac glucose uptake, we employed HL-1 cardiomyocytes in combination with transient siRNA-mediated gene knockdown (Fig. 4). Looking at transfection efficiency, siRNA treatment was effective in diminishing the protein expression of SNAP23, Cav-3, Stx7, and IRAP (Fig. S10). Depletion of SNAP23 largely decreased insulin-stimulated glucose uptake, ablating Δinsulin (Fig. 4A). Cav-3 knockdown induced an increase in basal glucose uptake and blunted insulin-stimulated glucose uptake (Fig. 4B). On the other hand, Cav-3 siRNA treatment did not affect Δinsulin in HL-1 cardiomyocytes (Fig. 4B) [48]. Knockdown of Stx7 or IRAP increased both basal and insulin-stimulated glucose uptake (Fig. 4C-D), but only IRAP knockdown increased Δinsulin (Fig. 4D). These data indicate that Cav-3, Stx7, and IRAP inhibit basal cardiac glucose uptake, with IRAP additionally inhibiting insulin-stimulated glucose uptake, while SNAP23 positively regulates insulin-stimulated glucose uptake. For SNAP23, this is in line with results in adipocytes [43, 44], and confirms that it also operates as t-SNARE in the heart. Conversely, the results with IRAP are not in line with the above-mentioned adipocyte silencing experiments [52] and point towards tissue-specific differences in GLUT4 traffic as further detailed in the discussion. Finally, Stx7 has never been associated before with glucose uptake regulation, while our data suggest that Stx7 mediates GLUT4 endocytosis.

### Lipid oversupply induces hyper-S-palmitoylation of Cav-3, Stx-7, and IRAP

**Fig. 5:**
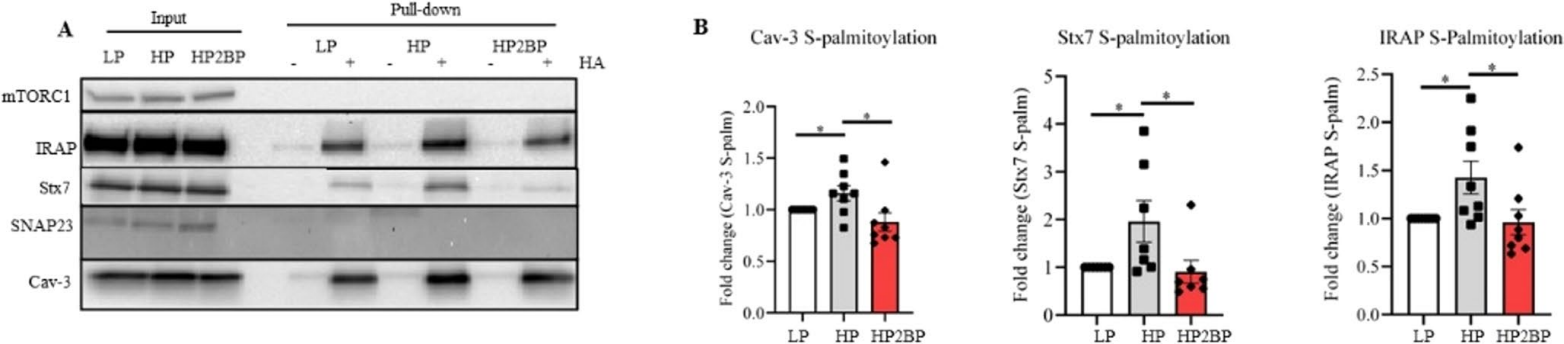
Acyl-biotinyl-exchange (ABE) to assess HP-induced changes in S-palmitoylation of proteins regulating glucose uptake in aRVCMs. Adult rat ventricular cardiomyocytes (aRVCMs) were cultured for 24 h in low palmitate (LP), high palmitate (HP), or high palmitate medium with 30 µM 2-bromopalmitate supplementation (HP2BP). Upon cell lysis, an ABE assay was performed. **A**-**B**) Representative blot (**A**) and quantification (**B**) of Cav-3 (*n* = 8), Stx7 (*n* = 7), and IRAP (*n* = 8) S-palmitoylation. Pull-downs [+ HA] (hydroxylamine) are normalized against inputs. mTORC1 is used as a negative control for S-palmitoylation. Data expressed as means ± SEM *p < 0.05 by two-tailed unpaired Student’s t-test. HA: hydroxylamine

Given that S-palmitoylation is substrate-driven, intracellular accumulation of palmitate is expected to increase protein S-palmitoylation [35]. We employed the ABE assay followed by SDS/PAGE-Western blot to assess whether HP exposure would increase S-palmitoylation of the selected proteins. mTORC1 is used as a negative control because it is known not to be S-palmitoylated in the heart [39]. Accordingly, it was also not picked up by our analysis of the cardiac S-palmitoylome (Table S1). Our findings indicate that HP exposure induced a significant increase in S-palmitoylation of Cav-3, Stx7, and IRAP in aRVCMs (Fig. 5A-B). The addition of 2BP to the HP-exposed cells prevented the increase in S-palmitoylation of these proteins (Fig. 5A-B). In our hands, despite being considered as a palmitoylated candidate from the palmitoylome analysis (Table S2), we could not detect any SNAP23 S-palmitoylation-band in the + HA fraction when performing Western blot after ABE assay (Fig. 5A). A possible explanation is that only a small portion of the total SNAP23 is palmitoylated in cardiomyocytes, which is below the threshold of detection of the SNAP23 antibody. Hence, further experiments with SNAP23 were abandoned. Taken together, we detected a hyper-S-palmitoylation of Stx7, Cav-3, and IRAP in aRVCMs upon lipid oversupply. Given that 2BP in lipid-overexposed cardiomyocytes preserved insulin-stimulated glucose uptake and contractility (Fig. 1), as well as restored normal-S-palmitoylation levels of all three selected proteins (Fig. 5), it seems feasible that beneficial 2BP effects are due to restricted S-palmitoylation of one (or more) of these proteins.

#### Cav-3 and IRAP are S-palmitoylated by DHHC5

**Fig. 6:**
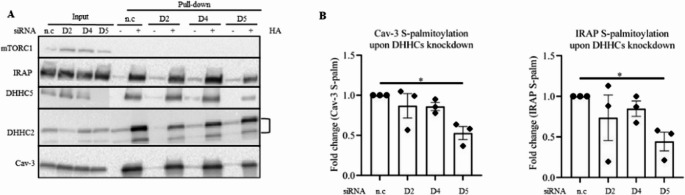
Unraveling specific DHHCs dedicated to S-palmitoylation of Cav-3 and IRAP in HL-1 cardiomyocytes. HL-1 cells were silenced with siRNAs against non-coding (n.c), DHHC2 (D2), DHHC4 (D4), and DHHC5 (D5) for 48 h in transfection medium, and subsequently incubated in control medium enriched with 20 µM palmitate (low palmitate) to induce basal palmitoylation. Upon cell lysis, an acyl-biotinyl exchange (ABE) assay was performed. **A**-**B**) Representative blot (**A**) and quantification (**B**) of IRAP and Cav-3 S-palmitoylation (*n* = 3). For DHHC2, two protein bands are detected. In order to visualize DHHC5 Input bands, the input lanes were derived from a blot with a relatively long exposure to the ECL kit, whereas the pull-down lanes were derived from the same blot with a relatively short exposure. Then all these lanes were merged into one image. Related to Fig. S14, which contains a representative DHHC5 blot with the short exposure time. mTORC1 immunoblot is the negative control for S-palmitoylation. Data expressed as means ± SEM *p < 0.05 by two-tailed unpaired Student’s t-test. HA: hydroxylamine

To determine which DHHC would be responsible for hyper-S-palmitoylation of Cav-3, Stx7, and IRAP, we performed siRNA-mediated knockdown of DHHC2, DHHC4, and DHHC5 in HL-1 cardiomyocytes and subsequently performed the ABE assay. As previously mentioned, silencing of DHHC4 greatly reduced *Dhhc4* expression levels compared to the control (Fig. S8C). Again, we confirmed silencing of DHHC2 and DHHC5 at the protein level (Fig. 6A). Accordingly, compared to the control, siRNA treatment decreased DHHC2 and DHHC5 auto S-palmitoylation (Fig. 6A), i.e., the first step by which the DHHCs catalyze S-palmitoylation of target proteins [53]. As also found in aRVCMs (Fig. 5), Cav-3 and IRAP were S-palmitoylated in HL-1 cells under basal conditions (Fig. 6). Unlike in aRVCMs, we could not detect any Stx7 band in [+ HA] in HL-1 cardiomyocytes (Fig. S11). S-palmitoylation of IRAP and Cav-3 were markedly decreased by DHHC5 knockdown, but not by DHHC2 or DHHC4. (Fig. 6). Altogether, among the three tested PATs, DHHC5 is the enzyme responsible for the S-palmitoylation of both IRAP and Cav-3.

We also assessed the interaction between DHHCs and their identified targets (IRAP/Cav-3) in co-immunoprecipitation (IP) experiments in aRVCMs. Our results show that both Cav-3 and IRAP interact with DHHC5, but not with DHHC2 (Fig. S12). Hence, the IP data are in line with the ABE data, further supporting the involvement of DHHC5, but not DHHC2, in S-palmitoylation of Cav-3 and IRAP. DHHC5 and GLUT4 are expressed in atrial vs. ventricular aRMCs almost equally (Fig. S13), compatible with S-palmitoylation similarly regulating glucose uptake in both tissues.

#### S-palmitoylation-deficient IRAP preserves insulin-stimulated glucose uptake and contractility

To further study the involvement of S-palmitoylation of Cav-3, Stx7, and IRAP in the onset of lipid-induced insulin resistance, we designed adenoviral vectors bearing a S-palmitoylation-deficient mutant version of each of these proteins (ΔCys constructs). The S-palmitoylated cysteines to be mutated within these proteins were selected based on the results of our MC-LS/LS analysis combined with Swiss-palm software (See Materials and Methods - Adenovirus construction and amplification).

Transductions of aRVCMs with WT Cav-3, Stx7, and IRAP adenoviral vectors and the respective ΔCys-Cav-3 (C19A, C106A, C116A, C129S, C140S), ΔCys-Stx7 (C28A, C239A), and ΔCys-IRAP (C103A, C114A) resulted in proper overexpression of these constructs and detection of the HA-tag (Fig. S15A-F). Fusion of Cav-3, Stx7, and IRAP with GFP in the adenoviral constructs allowed us to distinguish exogenous proteins from endogenous ones. We also established that the mutant proteins showed markedly reduced S-palmitoylation in aRVCMs compared to the WT counterparts (Fig. S15G-J).

**Fig. 7:**
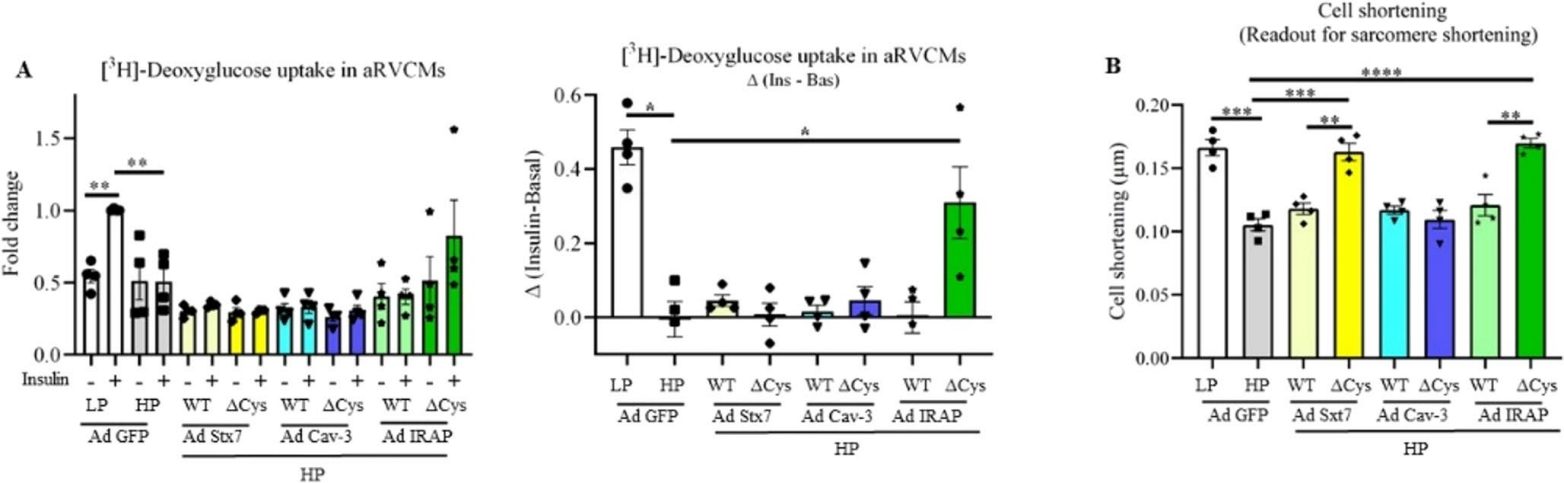
Overexpression of S-palmitoylation-deficient IRAP mutant (ΔCys-IRAP) in HP-treated aRVCMs restored insulin-stimulated glucose uptake and contractile dysfunction in lipid-overloaded aRVCMs. Adult rat ventricular cardiomyocytes (aRVCMs) were transduced with adenoviral vectors bearing GFP (control), WT syntaxin-7 (Stx7), WT caveolin-3 (Cav-3), WT IRAP and their according S-palmitoylation-deficient mutants (Cys◊Ala substitution; ΔCys) in low palmitate (LP) medium. After 24 h, cells were incubated for an additional 24 h in either LP or high palmitate (HP) medium. Glucose uptake and contractile function were assessed. (**A**) Quantification of [^3^H]-deoxyglucose uptake in aRVCMs transduced with different vectors and subsequently incubated for 30 min without (-) or with insulin (+, 100 nM) (*n* = 4). [^3^H]-deoxyglucose uptake values are displayed as fold changes normalized from the ‘LP + insulin’ condition’ (left panel), after which the Δinsulin was calculated (right panel). (**B**) Contractile function was assessed based on cell shortening (*n* = 4; imaging of 10 cells/measurement condition). Data expressed as means ± SEM *p < 0.05, **p < 0.01, ***p < 0.001, ****p < 0.0001 by two-tailed unpaired Student’s t-test.

The adenovirally transduced cells with WT and ΔCys forms of each of the selected proteins were used to measure insulin-stimulated glucose uptake and contractile activity. First, with respect to glucose uptake, insulin evokes a 2-fold increase in control cardiomyocytes cultured with LP medium. This effect of insulin is abolished in HP-exposed aRVCMs (Fig. 7A). Exogenous expression of WT proteins (Cav-3, Stx7, and IRAP) does not improve insulin-stimulated and Δinsulin glucose uptake, while ΔCys-IRAP mutant, but not ΔCys-Cav-3 and ΔCys-Stx7, significantly restored insulin-stimulated glucose uptake in HP-exposed aRVCMs (Fig. 7A). This suggests that among the selected proteins, IRAP hyper-S-palmitoylation is responsible for the inhibition of insulin-stimulated glucose uptake in palmitate-overloaded cardiomyocytes. We also measured the effects of the mutants on plasmalemmal CD36 levels. While short-term insulin in LP condition and long-term HP treatment in absence of insulin both induced CD36 translocation, the mutants did not reverse CD36 translocation or restore insulin-stimulated CD36 translocation in HP-exposed aRVCMs (Fig. S16A).

Next, video-based measurement of electrically-induced cell shortening (as readout of contractility of aRVCMs) revealed that HP induced a marked decrease in cell contractility (Fig. 7B; Fig. S16B), in line with Fig. 1G, in which sarcomere shortening was measured as readout of contractility. Exogenous expression of each of the WT proteins did not reverse the negative effect of HP on cell shortening. However, the ΔCys-Stx7 and ΔCys-IRAP mutants, but not ΔCys-Cav-3, completely prevented HP-induced contractile dysfunction (Fig. 7B). In conclusion, HP-induced hyper-S-palmitoylation of both Stx7 and IRAP could be attractive targets to restore contractile dysfunction in the lipid-overloaded heart. Remarkably, prevention of contractile dysfunction by the ΔCys-IRAP mutant, but not by the ΔCys-Stx7 mutant, was accompanied by preservation of insulin-stimulated uptake as further discussed below.

**Fig. 8:**
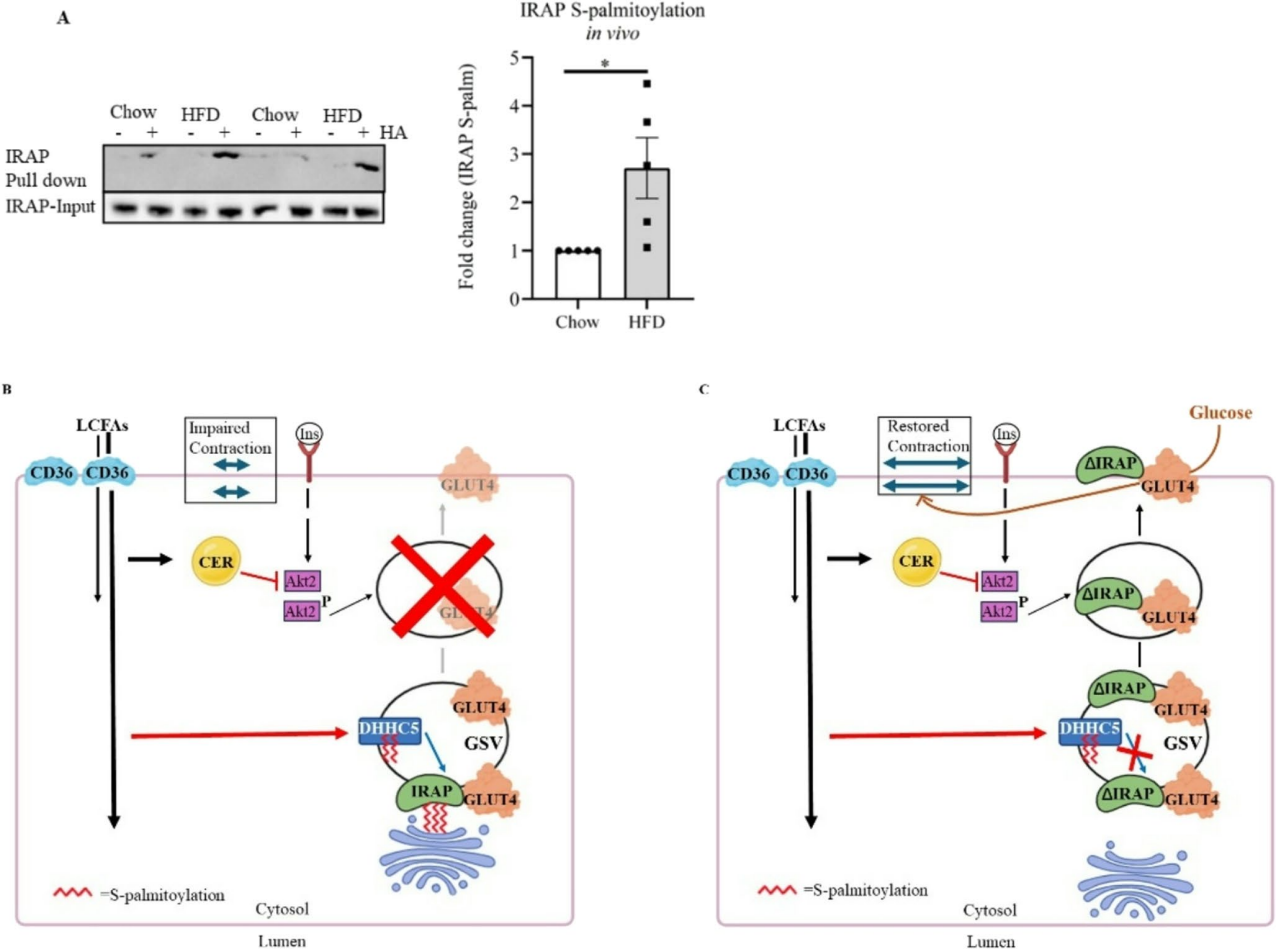
IRAP is hyper-S-palmitoylated in the lipid-overloaded heart in vivo. **A)** Snap-frozen hearts from control rats (Chow) and rats on a high-fat diet (HFD) were subjected to acyl resin–assisted capture for the purification of S-acylated proteins. The resulting IP fraction was used for Western blotting. (**A**) Representative blot and quantification of IRAP (*n* = 5) S-palmitoylation. Pull-downs [+ HA] (hydroxylamine) are normalized against inputs. Data expressed as means ± SEM *p < 0.05 by two-tailed unpaired Student’s t-test. HA: hydroxylamine. **B**,** C**): Mechanism by which IRAP hyper-palmitoylation contributes to lipid-induced insulin resistance in the heart. (**B**): Lipid overload: Long Chain Fatty Acids (LCFAs) oversupply results in increased LCFA uptake by CD36, which causes accumulation of triacylglycerol and lipid intermediates, such as ceramide (CER), partially impairing phosphorylation/activation of Akt2. Elevated LCFA flux also activates DHHCs, including DHHC5 (blue rectangle), which hyper-S-palmitoylates IRAP (green bean-shaped oval), resulting in anchoring of the GSVs to the Golgi. This completely inhibits GLUT4 translocation to the PM. The consequent inhibition of glucose uptake, combined with the initial increase in LCFA uptake, is a maladaptive deviation of the cardiac substrate balance, hence leading to contractile dysfunction. Importantly, limited Akt2 activation, albeit necessary for insulin-stimulated GLUT4 translocation, appears to be sufficient, thus rendering the partial CER-induced impairment without consequence on insulin-stimulated glucose uptake [54] (**C**): Lipid-overload & ΔCys-IRAP: The ΔCys-IRAP (ΔIRAP) mutant cannot be palmitoylated by DHHC5, and loses the ability to anchor the GSV to the Golgi. Hence, GLUT4 vesicles are liberated from their intracellular imprisonment and are able to translocate upon an insulin signal. As mentioned in the legend to panel B, the residual Akt2 activity (despite CER-induced impairment) is sufficient to allow full insulin-stimulated GLUT4 translocation, thereby increasing glucose uptake into the lipid-overloaded cardiomyocyte. The increased glucose flux then improves the cardiac substrate balance, and restores contractile force.

Finally, we investigated whether hyper-palmitoylation of IRAP also occurred in lipid-overloaded cardiomyocytes in vivo. For this, we used an established rodent model of hyperlipidemia [11, 28, 29]. Indeed, IRAP appeared to be hyper-palmitoylated 1.7-fold in lysates from diabetic hearts compared to control hearts (Fig. 8A), thereby adding evidence to the physiological relevance of this event upon lipid overconsumption.

## Discussion

The present study was designed to investigate the role of protein S-palmitoylation in the onset of CLIR. To our knowledge to date, no studies have yet explored the role of protein S-palmitoylation in cardiac insulin resistance. As hypothesized, lipid-overloaded cardiomyocytes exhibited increased protein S-palmitoylation. Inhibition of S-palmitoylation with the general inhibitor 2BP prevented key features of lipid-induced insulin resistance, including impaired insulin signaling, increased CD36 translocation, and reduced GLUT4-mediated glucose uptake [10, 36]. These effects were primarily attributed to DHHC5, which drove maladaptive S-palmitoylation. Palmitate oversupply also increased S-palmitoylation of Cav-3, Stx7, and IRAP, with DHHC5 mediating IRAP and Cav-3 hyper-S-palmitoylation. Genetic inhibition of IRAP S-palmitoylation prevented insulin resistance and contractile dysfunction, identifying a DHHC5–IRAP axis as a key pathway in lipid-overloaded hearts.

### Protein S-palmitoylation contributes to the onset of insulin resistance and contractile dysfunction

In our initial experiments, we observed that oversupply of palmitate induces general intracellular protein hyper-S-palmitoylation in aRVCMs and that co-treatment with 2BP restores S-palmitoylation to control (Fig. S2). Furthermore, we report here for the first time that co-treatment of palmitate-overloaded cardiomyocytes with 2BP prevents CD36 translocation to the plasma membrane, thus retaining CD36 intracellularly, and subsequently also averting the loss of GLUT4 translocation and insulin-stimulated glucose uptake in cardiomyocytes. Parts of these beneficial 2BP effects have been confirmed by another S-palmitoylation inhibitor (tunicamycin; Fig. S3), bolstering the notion that the 2BP effects are mediated via inhibition of S-palmitoylation, and not by an unknown off-target effect. This allows cardiomyocytes to retain a balanced uptake of the two major metabolic substrates (palmitate and glucose) under lipid oversupply conditions. This optimal balance between fatty acid and glucose uptake is necessary to maintain optimal cardiomyocyte contractile activity [2]. Accordingly, 2BP also offered protection against lipid-induced loss of contractile force, which implies that inhibition of (specific) S-palmitoylation may have the potential to offer a novel approach to protect the heart from lipid overload.

However, inhibiting general protein S-palmitoylation can cause unpredictable side effects, which makes 2BP less likely to be helpful in the clinic. Instead, targeting the S-palmitoylation enzyme isoforms DHHC2, DHHC4, and DHHC5 could be a more specific approach to restore insulin sensitivity in lipid-overloaded cardiomyocytes. Specifically, we demonstrated that knockdown of DHHC5, but not of DHHC2 or DHHC4, partially rescued insulin-stimulated glucose uptake. Moreover, DHHC5 inhibition also prevented CD36 translocation to the plasma membrane (Fig. S9), thereby replicating the beneficial actions of 2BP to counteract lipid accumulation in lipid-overloaded cardiomyocytes. This fits with the more general role of DHHC5 known to mediate S-palmitoylation proteins in subcellular/endosomal trafficking [55]. From a broader perspective and given that DHHC5 is among the most abundant PATs in the heart [56], pharmacological inhibition of DHHC5 may be a novel strategy to combat lipid-induced cardiomyopathy.

### S-palmitoylation of IRAP limits insulin-responsive cardiac glucose uptake upon lipid overload

Upon establishing protein S-palmitoylation involvement in the loss of insulin-stimulated glucose uptake in lipid-overloaded cardiomyocytes, we aimed to unveil which proteins involved in cellular glucose uptake are S-palmitoylated in the heart and, thereafter, to assess whether lipid (palmitate) oversupply induces changes in their S-palmitoylation. Analysis of the cardiac S-palmitoylome revealed several potential S-palmitoylated candidates: the t-SNAREs SNAP23 and Stx7, Cav-3, and the GLUT4 companion IRAP all negatively/positively regulate glucose uptake in cardiomyocytes (Fig. 4). We found that Cav-3, Stx7, and IRAP were hyper-palmitoylated in the HP condition (Fig. 5), which may suggest cellular mislocalization and impaired function of these proteins, as hyper-S-palmitoylation is generally associated with loss of protein function [23, 24]. Then, a combination of Mass spectrometry data and Swiss-palm (Fig. 3, Table S1-S2) software was used to mutate relevant Cys-residues in these three hyper-S-palmitoylated proteins to generate S-palmitoylation-deficient mutants. Of these three, only the IRAP mutant prevented palmitate-induced loss of insulin-stimulated glucose uptake and loss of contractile activity (Fig. 7A-B respectively). Moreover, IRAP is not only hyper-S-palmitoylated in vitro in HP-exposed aRVCMs, but also in vivo in a rat model of hyperlipidemia (Fig. 8A), suggesting that this event is physiological relevant during lipid-overconsumption. Accordingly, in the following paragraphs, we discuss the mechanism of the involvement of IRAP hyper-S-palmitoylation in lipid-induced contractile dysfunction in the context of the known functioning of IRAP in glucose uptake and GLUT4 traffic.

The GLUT4 travel companion IRAP resides in GSV [9, 50]. Its role in cardiac cells and, in particular, during lipid overload is less well studied. Previous findings have suggested that IRAP is involved in intracellular retention of GLUT4 within the GSVs [50, 57]. Herein, the cytosolic N-terminal plays an important role, as evidenced by increased plasma membrane GLUT4 localization in IRAP KO cells that is restored by the expression of the cytosolic domain of IRAP [9]. Likely, this N-terminal domain serves as an adaptor for cytosolic proteins, such as p115, tethering the GSV to the Golgi [50]. Accordingly, we observed increased glucose uptake upon IRAP knockdown in cardiomyocytes (Fig. 4).

As observed in HEK cells and adipocytes, IRAP is palmitoylated, but no role for this S-palmitoylation has been found so far [32, 33]. We found that DHHC5, but not DHHC2 or DHHC4, is specifically responsible for IRAP S-palmitoylation (Fig. 6). Moreover, we observed the occurrence of IRAP hyper-S-palmitoylation in lipid-overloaded cardiomyocytes (Fig. 5), which is likely mediated by DHHC5. The integration of DHHC5 and IRAP into a single pathway is further justified by the ABE experiments on lysates of HL-1 cells in which we knocked down the different DHHCs (Fig. 6), as well as in co-IP experiments (Fig. S12), showing that IRAP is a bona fide substrate of DHHC5.

Given that S-palmitoylation generally increases the interaction between proteins and intracellular membranes, it may be possible that the palmitate-induced increase in IRAP S-palmitoylation might favour the anchoring of the GSV to the Golgi, and thereby hinder GLUT4 translocation to the cell surface, thus inhibiting glucose uptake (Fig. 8B-C). The results with the ΔCys-IRAP mutant are entirely in line with this notion, given that this mutant, but not WT IRAP, protects against lipid-induced loss of insulin-stimulated glucose uptake. The resulting increase in glucose uptake may contribute to a re-balancing of cardiac substrate uptake, which is needed for optimal contractile activity of the cardiomyocytes [2, 36]. Indeed, the beneficial effect of the ΔCys-IRAP mutant on the re-instalment of insulin-stimulated glucose uptake was accompanied by a beneficial effect on cell shortening. However, a subsequent restoration of low levels of plasmalemmal CD36 has not (yet) occurred (Fig. S16A). Perhaps CD36 internalization would need longer time spans to develop following restoration of glucose uptake, because this would require a sufficient buildup of glucose intermediates over time to outcompete LCFA metabolism via Randle cycle effects [58] which then might impose feedback inhibition on CD36 trafficking via yet incompletely understood mechanisms. Hence, a partial restoration of the substrate balance suffices to restore cardiomyocyte contractility during lipid overload.

## Conclusions and future perspectives

By exploiting the tissue-specific nature of the S-palmitoylation machinery, the DHHC5-IRAP axis might hold promising therapeutic potential. For instance, in adipocytes, IRAP is palmitoylated by DHHC3 and, to a lesser extent, by DHHC7 [59]. However, DHHC3 is not expressed in cardiac muscle at the protein level [39]. Thus, additional investigations are necessary to assess whether DHHC5-mediated IRAP S-palmitoylation is uniquely restricted to the heart or is a mechanism that is also present in, e.g., skeletal muscle. If so, targeting the DHHC5–IRAP axis could not only repair the lipid-overloaded heart but also improve insulin resistance at the whole body level.

Finally, we wish to bring some loose ends to the attention. (i) We cannot entirely exclude a role for Cav-3 S-palmitoylation in lipid-induced insulin resistance. As mentioned, our ΔCys-Cav-3 construct preserved neither insulin-stimulated glucose uptake nor contractile function of the lipid-overloaded cardiomyocytes. Yet, this mutant was still palmitoylated (although reduced compared to WT - Fig. S15). Hence, other S-palmitoylation site(s), which are also substrate(s) for DHHC5, may still be involved. (ii) Another interesting finding was that the ΔCys-Stx7 mutant resolved lipid-induced contractile dysfunction independently of insulin-stimulated glucose uptake (Fig. 7), which deserves further studies. Investigating the role of Stx7 S-palmitoylation (besides GLUT4 translocation) in vesicular trafficking might be another line of research that could lead to novel therapeutic approaches against lipid-induced cardiomyopathy. (iii) This study has also led to the identification of the entire cardiac S-palmitoylome, which can be exploited by other researchers to check whether their protein of interest is S-palmitoylated, and hence subject to altered S-palmityolation-regulated dynamics.

## Supplementary Information

Below is the link to the electronic supplementary material.

Supplementary Material 1

## Data Availability

The data presented in this study are available upon request from the corresponding authors.
