# Supplementary material for: Lipid-induced S-palmitoylation of Insulin-Responsive Aminopeptidase (IRAP) drives the onset of insulin resistance in the heart
Source: Cell Mol Life Sci. 2026 Mar 26;83(1):187. doi: 10.1007/s00018-026-06179-0 (PMC13047037; doi:10.1007/s00018-026-06179-0)
Supplement: Supplementary file 1 — Supplementary Material 1 [file 18_2026_6179_MOESM1_ESM.docx]

**Lipid-induced S-palmitoylation of Insulin-Responsive Aminopeptidase (IRAP) drives the onset of insulin resistance in the heart**

Francesco Schianchi^1^, Jeroen Guns^2,3,4^, Freek G. Bouwman^5^, Jeroen F.J. Bogie^4^, Miranda Nabben^2,3,6^, Agnieszka Strzelecka^1^, Rick van Leeuwen^1,3^, Dimitris Kapsokalyvas^1,7^, Kaitlyn M.J.H. Dennis^8^, Lisa C. Heather^8^, Shujin Wang^1,9^, Jan F.C. Glatz^1,6^, Dietbert Neumann^3,10^, Joost J.F.P. Luiken^2,6^

^1^ Department of Genetics & Cell Biology, Maastricht University, 6229 ER Maastricht, The Netherlands.

^2^ Department of Cardiology, Maastricht University Medical Center+, Maastricht, The Netherlands.

^3^ CARIM School for Cardiovascular Diseases, Maastricht, The Netherlands.

^4^ Department of Immunology and Infection, Biomedical Research Institute, Hasselt University, Hasselt, Belgium.

^5^ Department of Human Biology; NUTRIM School of Nutrition and Translational Research in Metabolism; Faculty of Health, Medicine and Life Sciences, Maastricht, The Netherlands

^6^ Department of Clinical Genetics, Maastricht University Medical Center+, Maastricht, The Netherlands.

^7^ Interdisciplinary Centre for Clinical Research IZKF, University Hospital RWTH Aachen, 52074 Aachen, Germany.

^8^ Department of Physiology, Anatomy and Genetics, University of Oxford, United Kingdom.

^9^ Institute of Life Sciences, Chongqing Medical University, Chongqing, PR China.

^10^ Departments of Pathology, CARIM School for Cardiovascular Diseases, Maastricht University, Maastricht, The Netherlands

Dietbert Neumann and Joost J.F.P Luiken shared the last author

* Correspondence: Joost J.F.P. Luiken (J.J.F.P.L), PhD, Department of Cardiology, Maastricht University Medical Center+, P.O. Box 616, 6200 MD Maastricht, the Netherlands; E-mail: j.luiken@maastrichtuniversity.nl Telephone No: +31 641322401

Francesco Schianchi, PhD, Department of Genetics & Cell Biology, Maastricht University, 6229 ER Maastricht, The Netherlands; E-mail: schianchif90@hotmail.it; Telephone No: +31 645058179

**Supplementary figures**

**
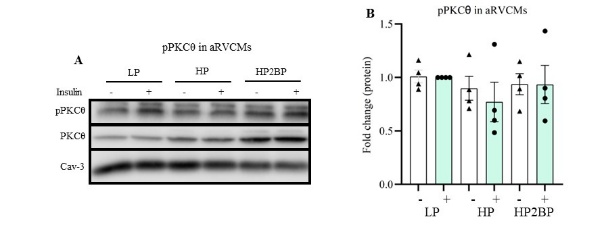
**

**Figure S1: Lipid-overload and S-palmitoylation did not alter pPKCθ phosphorylation in aRVCMs**. Adult rat ventricular cardiomyocytes (aRVCMs) were cultured for 24h in low (LP) or high palmitate (HP) medium, treated without or with 30 µM 2-bromopalmitate (2BP) supplementation (HP2BP). Thereafter, cells were treated for 30 min without (-, white bars) or with insulin (+, light green bars, 100 nM). **A,B**) Representative blot (**A**) and quantification of pPKCθ (**B**) protein expression in aRVCMs treated with different conditions (n=4) as measurement for insulin sensitivity. pPKC was normalized against total PKC and further normalized to Caveolin 3 (Cav-3) content which was used as loading control. Data expressed as means ± SEM *p<0.05, **p<0.01, ***p<0.001, ****p<0.0001 by two-tailed unpaired Student’s t-test.

**
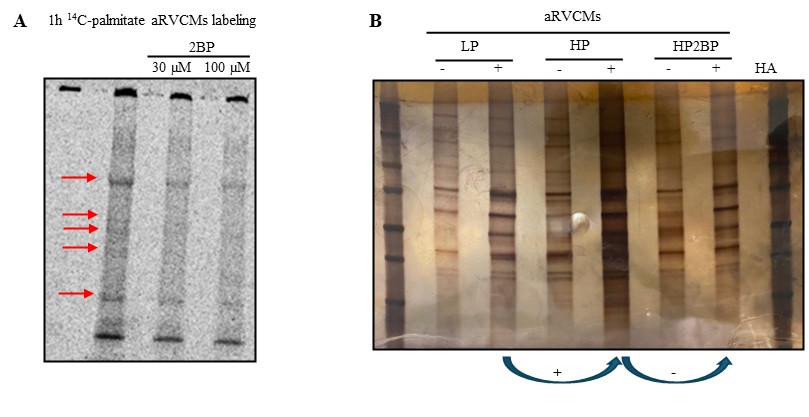
**

**Figure S2: Verification of 2-bromopalmitate (2BP) as general S-palmitoylation inhibitor.**

**A)** Autoradiogram of Adult rat ventricular cardiomyocytes (aRVCMs) cultured for 1h with 7 μCi ^14^C-palmitate complexed with α-cyclodextrin without or with 30 or 100 µM 2-bromopalmitate supplementation (2BP) in low palmitate (LP) medium. **B)** Silver staining of aRVCMs cultured for 24h in LP, high palmitate (HP), or HP medium with 30 µM 2-bromopalmitate supplementation (HP2BP). Upon cell lysis, an ABE assay was performed. HA: hydroxylamine.

**
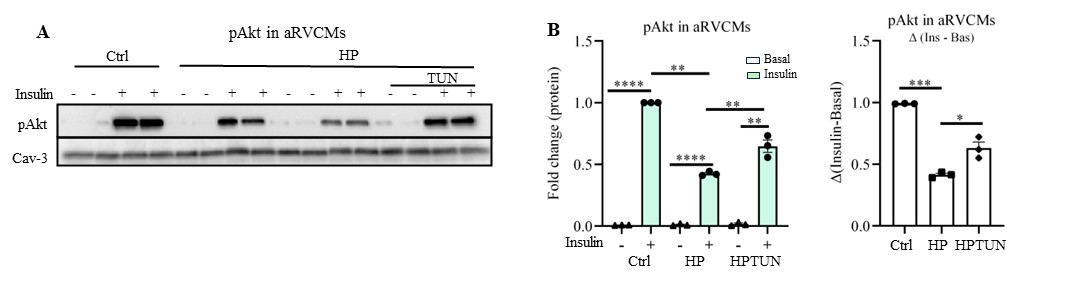
**

**Figure S3: S-palmitoylation impairs insulin sensitivity in aRVCMs.**

Adult rat ventricular cardiomyocytes (aRVCMs) were cultured for 24h in low (LP) or high palmitate (HP) medium, treated without or HP medium with 3 µM tunicamycin (TUN) supplementation (HPTUN), and subsequently incubated for 30 min without (-, white bars) or with insulin (+, light green bars, 200 nM). **A-B)** Representative blot (**A**) and quantification (**B**) of pAkt Ser473 in aRVCMs treated under different conditions as a measure of insulin sensitivity. Caveolin-3 (Cav-3) was used as a loading control. (n=3). The insulin effect (ΔInsulin**;** right graph) is displayed for each condition, representing the calculated difference (Δ) between - and + insulin stimulation. Data expressed as means ± SEM *p<0.05, **p<0.01, ***p<0.001, ****p<0.0001 by two-tailed unpaired Student’s t-test. Note that in this blot the “HP” condition was loaded twice.


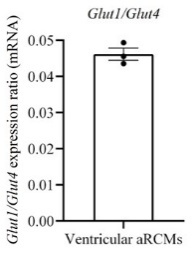


**Figure S4: Expression of GLUT4 exceeds that of GLUT1 in ventricular aRVCMs.**

Ratio of RT-qPCR of *Glut1/Glut4* gene expression in ventricular cardiomyocytes in LP medium (n=3).

**
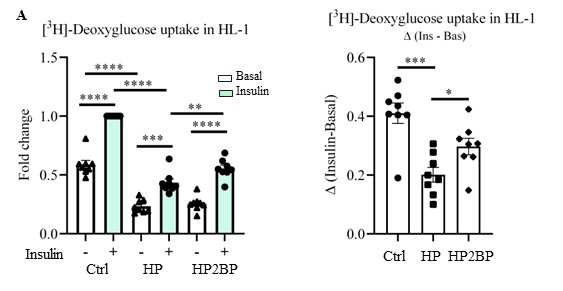
**

**Figure S5: S-palmitoylation impairs insulin-stimulated glucose uptake in high palmitate-treated cardiomyocytes.**

HL-1 cells were incubated overnight in control (Ctrl), high palmitate (HP) medium, or HP medium with 30 µM 2-bromopalmitate supplementation (HP2BP) and subsequently incubated for 30 min without (-, white bars) or with insulin (+, light green bars, 200 nM). Quantification of [^3^H]-deoxyglucose uptake in HL-1 cells treated under different conditions (n=8). [^3^H]-deoxyglucose uptake values are displayed as fold changes normalized from the ‘n.c. + insulin’ condition’ (left panels), after which the Δinsulin was calculated (right panels). Data expressed as means ± SEM *p<0.05, ***p<0.001, ****p<0.0001 by two-tailed unpaired Student’s t-test.


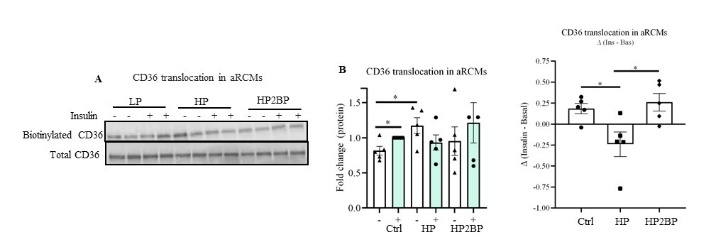


**Figure S6: 2BP restored insulin-stimulated CD36 translocation in palmitate-overloaded aRVCMs.**

Adult rat ventricular cardiomyocytes (aRVCMs) were cultured for 24h in low (LP) or high palmitate (HP) medium, treated without or with 30 µM 2-bromopalmitate (2BP) supplementation (HP2BP). Thereafter, cells were treated for 30 min without (-, white bars) or with insulin (+, light green bars, 100 nM). Cell surface CD36 translocation was assessed via biotin-immunoprecipitation assay.

Representative blot (A) and quantification (b) of CD36 biotin-immunoprecipitation and total lysate fractions in aRVCMs treated with different conditions (n=5). The insulin effect (ΔInsulin**;** right graph) is displayed for each condition, representing the calculated difference (Δ) between - and + insulin stimulation. Data expressed as means ± SEM *p<0.05, **p<0.01, ***p<0.001, ****p<0.0001 by two-tailed unpaired Student’s t-test.

**
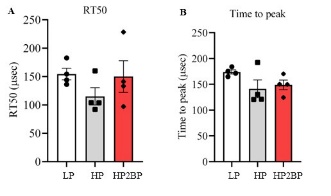
**

## **Figure S7: Effect of 2BP on RT50 and Time to peak in palmitate-overloaded aRVCMs.**

## Adult rat ventricular cardiomyocytes (aRVCMs) were cultured for 24h in low palmitate (LP), high palmitate (HP), or high palmitate medium with 30 µM 2-bromopalmitate supplementation (HP2BP). Thereafter, contractile parameters (A) decay time to 50% (RT50), and (B) time to peak were measured (n=4). Imaging of 10 cells measurement/condition.

**
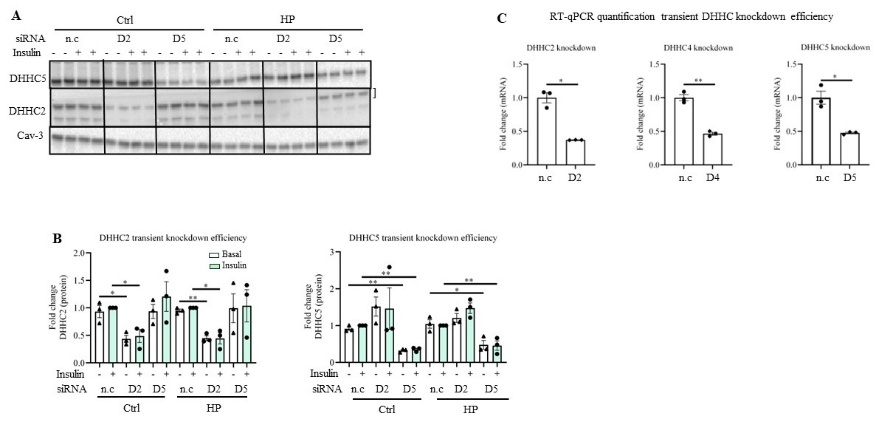
**

**Figure S8:** **DHHC2, DHHC4, and DHHC5 knockdown in HL-1 cardiomyocytes.**

HL-1 cells were silenced with siRNAs against non-coding (n.c), DHHC2 (D2), DHHC4 (D4), and DHHC5 (D5) for 48h in transfection medium, then stimulated for 16h with control (Ctrl) or high palmitate (HP) medium, and subsequently incubated for 30 min without (-, white bars) or with insulin (+, light green bars, 200 nM). **A-B)** Representative blot (A) and quantification (B) of DHHC5 and DHHC2 protein expression in HL-1 transfected with different siRNAs. Upon transfection, and 16h incubation with the respective medium, cells were subjected to short-term (30 min; 200 nM) insulin stimulation and lysed (n=3). Caveolin-3 (Cav-3) was used as a loading control. White bars: no stimulation (- insulin); light green bars: stimulation with insulin (+ insulin). **C)** RT-qPCR of *DHHC2, DHHC4,* and *DHHC5* gene expression after siRNA transfection in Ctrl medium (n=3). Data expressed as means ± SEM *p<0.05, **p<0.01 by two-tailed unpaired Student’s t-test.


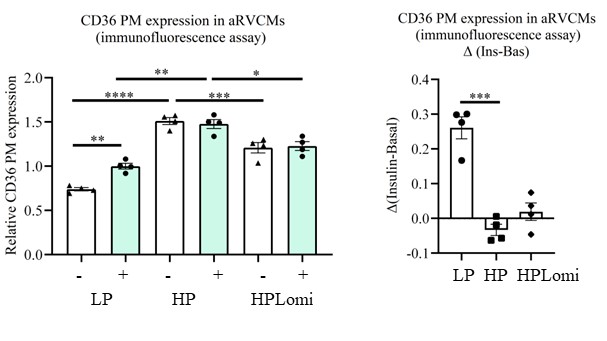


**Figure S9:** **DHHC5 mediates increased CD36 translocation in HP-exposed aRVCMs.**

Adult rat ventricular cardiomyocytes (aRVCMs) were cultured for 24h in low (LP) or high palmitate (HP) medium, treated without or with 1 µM DHHC5 inhibitor lomitapide (Lomi) supplementation (HPLomi). Thereafter, cells were treated for 30 min without (-, white bars) or with insulin (+, light green bars, 100 nM). CD36 plasma membrane (PM) expression in aRVCM was assessed by immunofluorescence assay with fluorescent-labelled CD36 antibody (n=4). CD36 PM expression values are displayed as fold changes normalized from the ‘LP + insulin’ condition (left panel), after which the Δinsulin was calculated (right panel).

Data expressed as means ± SEM *p<0.05, **p<0.01 by two-tailed unpaired Student’s t-test.


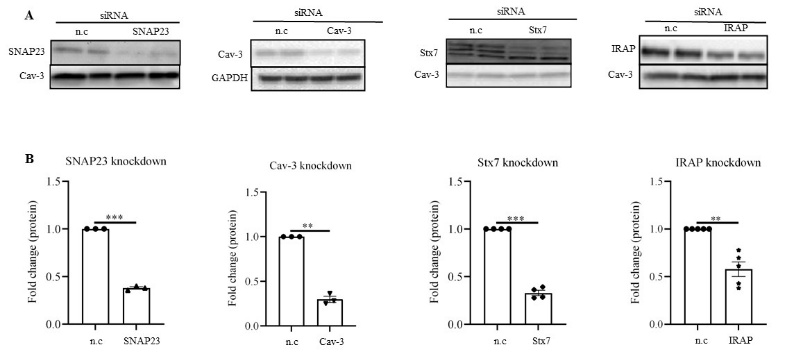


**Figure S10:** **Efficiency of SNAP23, Cav-3, Stx7, and IRAP, Stx7 transient knockdown in HL-1 cardiomyocytes.**

HL-1 cells were silenced with siRNAs against non-coding (n.c), SNAP23, caveolin-3 (Cav-3), syntaxin-7 (Stx7), and IRAP for 48h in control (Ctrl) medium. **A-B)** Representative blot (A) and quantification (B) of SNAP23 (n=4), Cav-3 (n=3), Stx7 (n=4), and IRAP (n=5) protein expression in HL-1 cells silenced for the different genes. Cav-3 or GAPDH was used as a loading control. Data expressed as means ± SEM **p<0.01, ***p<0.001 by two-tailed unpaired Student’s t-test.

**
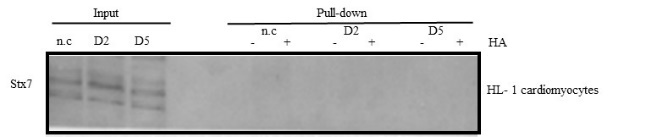
**

**Figure S11:** **Absence of** **Stx7 S-palmitoylation in HL-1 cardiomyocytes**.

HL-1 cells were silenced with siRNAs against non-coding (n.c), DHHC2 (D2), or DHHC5 (D5) for 48h in transfection medium and subsequently incubated for 1h in control (Ctrl) medium enriched with 20 µM palmitate. Upon cell lysis, an ABE assay was performed. Representative blot of Stx7 S-palmitoylation upon silencing of D2 or D5 (n=3). The enriched-S-palmitoylated Stx7 bands were not detected, indicating the absence of Stx7 S-palmitoylation in HL-1 cardiomyocytes. HA: hydroxylamine.

## **
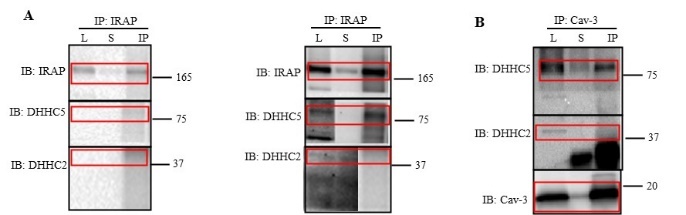
**

## **Figure S12: Assessing interaction of IRAP and Cav-3 with DHHC2 and DHHC5 via immunoprecipitation.**

## Adult rat ventricular cardiomyocytes (aRVCMs) were cultured for 24h in palmitate (LP) medium. The interaction of several DHHCs with IRAP and caveolin-3 was assessed. A-B) Immunoprecipitation of IRAP (A) and Cav-3 (B) with DHHC2 and DHHC5 in aRVCMs (n=1). L: lysate; S: supernatant of the IP; IP: immunoprecipitation; IB: immunoblot.

## **
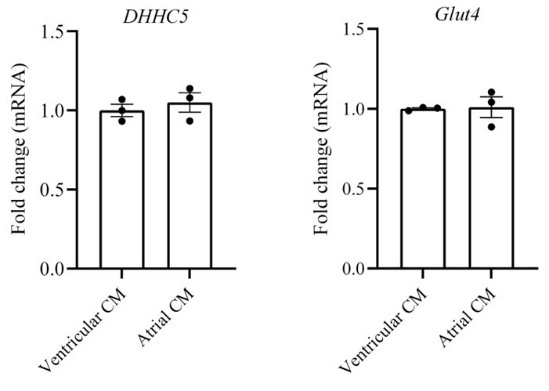
**

**Figure S13:** RT-qPCR of *Dhhc5,* and *Glut4* gene expression in both ventricular and atrial cardiomyocytes in LP medium (n=3). Data expressed as means ± SEM *p<0.05, **p<0.01 by two-tailed unpaired Student’s t-test.


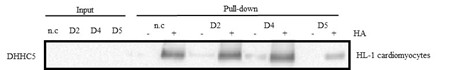


## **Figure S14: Original DHHC5 blot upon DHHC5 pull-down belonging to Fig. 7.**

## HL-1 cells were silenced with siRNAs against non-coding (n.c), DHHC2 (D2), DHHC4 (D4), and DHHC5 (D5) for 48h in transfection medium, and subsequently incubated in control medium enriched with 20 µM palmitate (low palmitate) to induce basal palmitoylation. Upon cell lysis, an acyl-biotinyl exchange (ABE) assay was performed. Presentation of the original blot related to Figure 6 with short ECL exposure, which only allowed visualization of enrichment of S-palmitoylated DHHC5. HA: hydroxylamine.

**
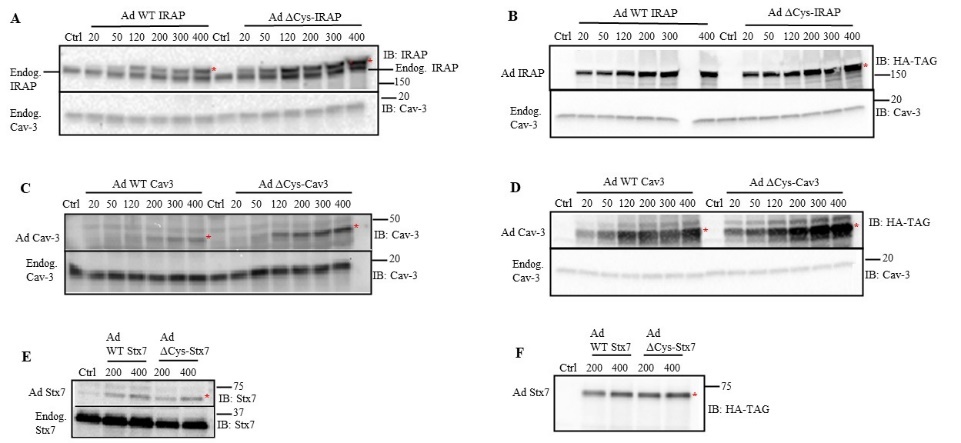
**

**
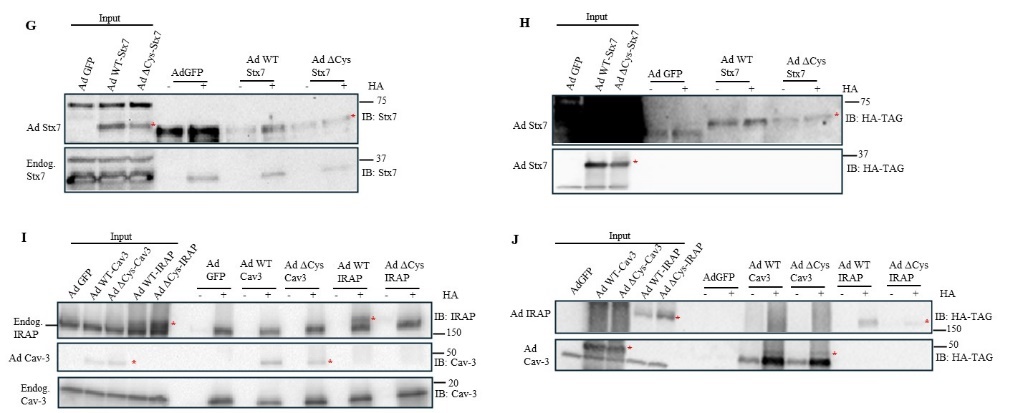
**

**Figure S15:** **Verification of overexpression and loss of S-palmitoylation after transduction IRAP/Cav-3/Stx7 Cys mutants in aRVCMs.**

Adult rat ventricular cardiomyocytes (aRVCMs) were transduced with adenoviral vectors bearing GFP (control), WT syntaxin-7 (Stx7), WT caveolin-3 (Cav-3), WT IRAP and their corresponding S-palmitoylation-deficient mutants (Cys🡪Ala substitution; ΔCys) in low palmitate (LP) medium. Upon cell lysis, protein expression was assessed with Western blot or an ABE was performed. **A-F)** Representative blots of IRAP (**A-B**), Cav-3 (**C-D**), and Stx7 (**E-F**) protein expression upon specific viral transduction. Overexpression has been verified by antibodies against native protein (A**,C,E**) and against HA-tag (**B,D,F**). **G-J)** S-palmitoylation of WT and mutant forms has been assessed by IP in combination with ABE (-HA/+HA) by antibodies against native protein (**G, I**) or against HA-tag (**H, J**). *=Protein bands of the constructs are marked with * to distinguish the exogenous proteins from the endogenous ones. HA: hydroxylamine.


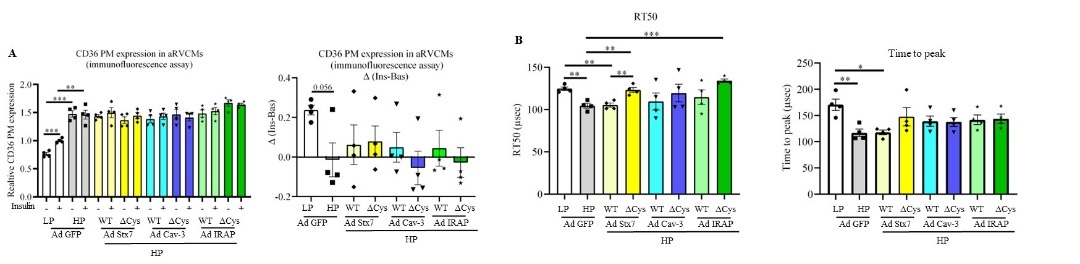


**Figure S16: Effects of S-palmitoylation-deficient mutants on plasma membrane (PM) expression of CD36 and kinetic contractile parameters in HP-treated aRVCMs.**

Adult rat ventricular cardiomyocytes (aRVCMs) were transduced with adenoviral vectors bearing GFP (control), WT syntaxin-7 (Stx7), WT caveolin-3 (Cav-3), WT IRAP and their according S-palmitoylation-deficient mutants (Cys🡪Ala substitution; ΔCys) in low palmitate (LP) medium. After 24h, cells were incubated for an additional 24h in either LP (LP) or high palmitate (HP) medium. CD36 plasma membrane (PM) expression and contractile parameters were assessed based on decay time to 50% (RT50), and time to peak. **(A)** After 24h incubation in LP or HP, cells were treated for 30 min without (-) or with insulin (+, 100 nM). CD36 plasma membrane (PM) expression in aRVCM was assessed by immunofluorescence assay with fluorescent-labelled CD36 antibody (n=4). CD36 PM expression values are displayed as fold changes normalized from the ‘LP + insulin’ condition (left panel), after which the Δinsulin was calculated (right panel).

**(B)** After 24h incubation in LP or HP, decay time to 50% (RT50 left panel) and time to peak (TTP; right panel) were assessed (n=4; imaging of 10 cell measurements/condition). Data expressed as means ± SEM *p<0.05, **p<0.01, ***p<0.001, ****p<0.0001 by two-tailed unpaired Student’s t-test.

## **Supplementary Table S1**. Proteins identified in rat cardiomyocytes palmitoylome, with statistics of proteomics.

| **Accession**  **number** | **Protein name** | **[+HA]/[-HA]**  **ΣhMS1 ratio** |
| --- | --- | --- |
| Q10743 | Disintegrin and metalloproteinase domain-containing protein 10 | 384,425 |
| P97687 | Ectonucleoside triphosphate diphosphohydrolase 1 | 353,5666667 |
| Q2IBC5 | Caveolin-2 | 347,61 |
| Q62786 | prostaglandin F2 receptor negative regulator | 340,16 |
| Q5FVQ4 | Malectin | 327,35 |
| Q9Z1W6-1 | protein LYRIC | 318,53 |
| Q62745 | CD81 antigen | 312,35 |
| Q9Z2Q7 | Syntaxin-8 | 300,4694412 |
| O70257 | Syntaxin-7 | 298,7166667 |
| Q6AY20 | Cation-dependent mannose-6-phosphate receptor | 288,1125 |
| Q5BJK8 | Golgi integral membrane protein 4 | 282,6125 |
| Q9EPF2-1 | Cell surface glycoprotein MUC18 | 282,5224242 |
| Q6IMY1-1 | Microtubule-associated tumor suppressor 1 homolog | 276,6625 |
| G3V7P1 | Syntaxin-12 | 275,67 |
| Q6AYC2 | Immunity-related GTPase family M protein | 274,2833333 |
| P40241 | CD9 antigen | 269,5310734 |
| Q5XIB3 | [Protein ADP-ribosylarginine] hydrolase-like protein 1 | 266,9862282 |
| O70377 | Synaptosomal-associated protein 23 | 264,4277778 |
| P82471 | Guanine nucleotide-binding protein G(Q) subunit alpha | 260,1291616 |
| Q9JID2 | guanine nucleotide-binding protein subunit alpha-11 | 254,5713725 |
| Q9R066 | Coxsackievirus and adenovirus receptor homolog | 253,5962712 |
| Q6P791 | ragulator complex protein LAMTOR1 | 247,1375 |
| P60905 | DnaJ homolog subfamily C member 5 | 235,9874631 |
| P27615 | Lysosome membrane protein 2 | 226,6325495 |
| Q9QXU2 | Surfeit locus protein 1 | 211,0834802 |
| P01112 | RASH_HUMAN | 209,3405522 |
| P97629 | Leucyl-cystinyl aminopeptidase | 201,0506008 |
| P49242 | 40S ribosomal protein S3a | 200,338302 |
| P41350-1 | Caveolin-1 | 195,8044658 |
| O88483 | [Pyruvate dehydrogenase [acetyl-transferring]]-phosphatase 1, mitochondrial | 192,6578348 |
| Q99M64 | Phosphatidylinositol 4-kinase type 2-alpha | 192,610101 |
| P84060 | Dystrobrevin beta | 188,1290207 |
| P56603 | Secretory carrier-associated membrane protein 1 | 186,6700097 |
| B5DFN3 | Ubiquinol-cytochrome-c reductase complex assembly factor 2 | 185,4035704 |
| Q5U1Z2 | Trafficking protein particle complex subunit 3 | 177,664214 |
| A2VD12 | Pre-B-cell leukemia transcription factor-interacting protein 1 | 168,1705263 |
| B4F795 | Choline transporter-like protein 2 | 167,7828447 |
| B0BN86 | Transmembrane protein 11, mitochondrial | 166,9379509 |
| Q6Q7Y5 | Guanine nucleotide-binding protein subunit alpha-13 | 166,2081715 |
| Q2PS20 | Junctophilin-2 | 165,5838601 |
| Q63258 | integrin alpha-7 | 142,5038817 |
| Q6VBQ5 | myeloid-associated differentiation marker | 141,4837703 |
| P59215-2 | Isoform Alpha-2 of Guanine nucleotide-binding protein G(o) subunit alpha | 137,0123249 |
| P47727 | Carbonyl reductase [NADPH] 1 | 134,4870974 |
| P27605 | Hypoxanthine-guanine phosphoribosyltransferase | 134,1017312 |
| B0LPN4 | ryanodine receptor 2 | 134,0318749 |
| Q68FU7 | Ubiquinone biosynthesis monooxygenase COQ6, mitochondrial | 133,7875233 |
| P02770 | Serum albumin | 133,7452862 |
| A2RRU1 | glycogen [starch] synthase, muscle | 133,5947684 |
| Q6AXT8 | splicing factor 3a subunit 2 | 133,577554 |

**Table S1** *Continued*

| **Accession**  **number** | **Protein name** | **[+HA]/[-HA]**  **ΣhMS1 ratio** |
| --- | --- | --- |
| P63095 | Guanine nucleotide-binding protein G(s) subunit alpha isoforms short | 126,4911974 |
| Q91XR8 | Phospholipid hydroperoxide glutathione peroxidase, nuclear | 116,2882626 |
| D3Z8L7 | Ras-related protein R-Ras | 112,9304285 |
| Q01728-7 | Isoform 7 of Sodium/calcium exchanger 1 | 110,9773848 |
| Q01728 | Sodium/calcium exchanger 1 | 106,173451 |
| Q64536 | [Pyruvate dehydrogenase (acetyl-transferring)] kinase isozyme 2, mitochondrial | 104,3071437 |
| Q9WUH4 | Four and a half LIM domains protein 1 | 100,8785468 |
| O89049 | Thioredoxin reductase 1, cytoplasmic | 100,8579399 |
| P48675 | desmin | 100,7175634 |
| O88600 | Heat shock 70 kDa protein 4 | 100,5457809 |
| P63245 | Receptor of activated protein C kinase 1 | 100,3204349 |
| P35565 | Calnexin | 99,09268076 |
| Q5PPN7 | Coiled-coil domain-containing protein 51 | 94,25709887 |
| B2GV54 | Neutral cholesterol ester hydrolase 1 | 90,23096952 |
| Q9Z1E1-1 | Flotillin-1 | 89,38301629 |
| Q07803 | Elongation factor G, mitochondrial | 88,66302412 |
| Q62638 | Golgi apparatus protein 1 | 85,27874546 |
| Q99MI7 | NEDD8-activating enzyme E1 catalytic subunit | 83,78090852 |
| P36201 | Cysteine-rich protein 2 | 81,3624218 |
| Q6IG05 | Keratin, type II cytoskeletal 75 | 81,20105376 |
| O55171 | Acyl-coenzyme A thioesterase 2, mitochondrial | 81,12135332 |
| Q9QZ76 | Myoglobin | 81,07442531 |
| Q10758 | Keratin, type II cytoskeletal 8 | 81,03879475 |
| Q6IFU7 | Keratin, type I cytoskeletal 42 | 80,96201701 |
| P62632 | Elongation factor 1-alpha 2 | 80,4819432 |
| B2RZ37 | Receptor expression-enhancing protein 5 | 75,52816899 |
| P57093 | Phytanoyl-CoA dioxygenase, peroxisomal | 48,71607683 |
| P29266 | 3-hydroxyisobutyrate dehydrogenase, mitochondrial | 48,47465291 |
| Q9ESS6 | Basal cell adhesion molecule | 46,81096681 |
| P51638 | Cav-3 | 41,22325874 |
| P06686 | Sodium/potassium-transporting ATPase subunit alpha-2 | 37,28414358 |
| O35567 | bifunctional purine biosynthesis protein purH | 35,36508497 |
| P04897 | guanine nucleotide-binding protein G(i) subunit alpha-2 | 30,49915993 |
| Q07969 | Platelet glycoprotein 4 | 28,50017927 |
| P04041 | Glutathione peroxidase 1 | 28,42780687 |
| Q9Z2S9 | Flotillin-2 | 28,33519641 |
| P35738 | 2-oxoisovalerate dehydrogenase subunit beta, mitochondrial | 27,55605511 |
| P08461 | Dihydrolipoyllysine-residue acetyltransferase component of pyruvate  dehydrogenase complex, mitochondrial | 18,51406848 |
| Q09073 | ADP/ATP translocase 2 | 15,04313535 |
| Q9EQX9 | ubiquitin-conjugating enzyme E2 N | 13,68801428 |
| Q642A4 | UPF0598 protein C8orf82 homolog | 13,37129647 |
| Q920F5-1 | Malonyl-CoA decarboxylase, mitochondrial | 13,30364895 |
| P61626 | LYSC_HUMAN | 12,58176358 |
| Q63065 | [Pyruvate dehydrogenase (Acetyl-transferring)] kinase isozyme 1, mitochondrial | 11,97857465 |
| P17764 | Acetyl-CoA acetyltransferase, mitochondrial | 9,985694129 |
| Q8K4G6 | O-acetyl-ADP-ribose deacetylase MACROD1 | 7,044373053 |
| Q5BJQ0 | Atypical kinase ADCK3, mitochondrial | 6,784514785 |
| Q02253 | Methylmalonate-semialdehyde dehydrogenase [acylating], mitochondrial | 6,737305789 |
| P97576 | GrpE protein homolog 1, mitochondrial | 5,853333052 |

**Table S1** *Continued*

| **Accession**  **number** | **Protein name** | **[+HA]/[-HA]**  **ΣhMS1 ratio** |
| --- | --- | --- |
| Q60587 | Trifunctional enzyme subunit beta, mitochondrial | 5,44607477 |
| Q01205 | Dihydrolipoyllysine-residue succinyltransferase component of 2-oxoglutarate  dehydrogenase complex, mitochondrial | 5,382015983 |
| P07633 | propionyl-CoA carboxylase beta chain, mitochondrial | 4,716387112 |
| P23693 | Troponin I, cardiac muscle | 4,697754242 |
| P26453-1 | Basigin | 4,451431859 |
| Q5U300 | Ubiquitin-like modifier-activating enzyme 1 | 3,970684761 |
| Q8VBU2 | Protein NDRG2 | 3,575529298 |
| Q9R063-1 | Peroxiredoxin-5, mitochondrial | 3,532137342 |
| P97521 | mitochondrial carnitine/acylcarnitine carrier protein | 3,504731235 |
| Q9WVK7 | Hydroxyacyl-coenzyme A dehydrogenase, mitochondrial | 3,11620621 |
| P62839 | ubiquitin-conjugating enzyme E2 D2 | 3,069384448 |
| P13795-1 | Synaptosomal-associated protein 25 | 3,066551607 |
| Q9R1Z0-2 | Isoform 2 of Voltage-dependent anion-selective channel protein 3 | 3,00115367 |
| P56571 | ES1 protein homolog, mitochondrial | 2,874702533 |
| Q5XI32 | F-actin-capping protein subunit beta | 2,65221733 |
| P07340 | Sodium/potassium-transporting ATPase subunit beta-1 | 2,565561102 |
| B0K020 | CDGSH iron-sulfur domain-containing protein 1 | 2,554550797 |
| Q06647 | ATP synthase subunit O, mitochondrial | 2,384316789 |
| Q9Z0V6 | Thioredoxin-dependent peroxide reductase, mitochondrial | 2,363667749 |
| P14604 | Enoyl-CoA hydratase, mitochondrial | 2,3353063 |
| B2RYW9 | Fumarylacetoacetate hydrolase domain-containing protein 2 | 2,323230881 |
| P11884 | Aldehyde dehydrogenase, mitochondrial | 2,245819389 |
| D3ZDK2 | Ubiquitin-conjugating enzyme E2 D1 | 2,240737419 |
| Q6AXV4 | sorting and assembly machinery component 50 homolog | 2,224701187 |
| P81155 | Voltage-dependent anion-selective channel protein 2 | 2,214601305 |
| P21913 | Succinate dehydrogenase [ubiquinone] iron-sulfur subunit, mitochondrial | 2,212702092 |
| Q07936-2 | Isoform Long of Annexin A2 | 2,184035655 |
| Q499N5 | Acyl-CoA synthetase family member 2, mitochondrial | 2,182395386 |
| P35434 | ATP synthase subunit delta, mitochondrial | 2,13798147 |
| P26284 | Pyruvate dehydrogenase E1 component subunit alpha, somatic form,  mitochondrial | 2,067348697 |
| P08733 | myosin regulatory light chain 2, ventricular/cardiac muscle isoform | 2,024803077 |
| D3ZAF6 | ATP synthase subunit f, mitochondrial | 2,011376586 |
| P53987 | Monocarboxylate transporter 1 | 2,010928227 |

## Accession number: protein accession number in Uniprot.

## ΣhMS1: sum of the height of the peaks of all the detected unique peptides (ΣhMS1) for a given protein.

## [+HA]/[-HA] ΣhMS1 ratio: ΣhMS1 in +HA fraction were plotted against ΣhMS1 in -HA fraction (negative control). Proteins having a [+HA]/[-HA] ΣhMS1 ratio >2 are presented in the table. A ratio greater than 2 indicates that the given entry is likely to be a palmitoylated protein. Each protein is color-paired to its respective protein class (**Fig. 3D**)

| **Palmitolyated proteins found enriched in +HA**  **[+HA]/[-HA] ΣhMS1 ratio >2** | **Cys residue(s)**  **found by Mass-Spec analysis**  **Enriched**  **In + HA fraction** | **SwissPalm**  **Prediction** | **Literature** | **Function of**  **S-palmitoylation** |
| --- | --- | --- | --- | --- |
| **SNAP23:**  Regulates GLUT4 trafficking | None | 79,80,83,85,87 | V. Agarwa *et al.,* 2019,  (Cys 79,80,83,85,87 identified) [43] | Necessary for plasma  membrane association  of SNAP23 (tested in synthetic vesicles in cell-free systems) [43] |
| **Syntaxin-7 (Stx7):**  Might be involved in endosome-plasma membrane trafficking (GLUT4?) | 28 | None | Yuhong He and Maurine E. Linder., 2009 (Cys 239 identified) [28] | Unknown |
| **Caveolin-3 (Cav-3):**  Plasma membrane protein interacting with Insulin Receptor | 19, 129 and 140 | Cys 19, 106, 116, 129 (Medium confidence prediction) | W. Ren *et al.,* 2016  Adipocytes (No Cys identified) [21]  Badrilla Acyl Peg Kit identified 5 Cys (19, 106,119 129, 140)  [Acyl Peg Exchange – an important Advance in the S-palmitoylation Toolbox – Badrilla] | Unknown |
| **IRAP:**  Translocates with GLUT4 from GLUT4 storage compartments to the plasma membrane | 103 | None | Martin W. Werno & Luke H. Chamberlain., 2015 (Cys 103, 114 identified) [30] | Unknown |

**Supplementary Table S2.** Table summarizing the S-palmitoylated candidates of interest and their known role in glucose uptake.
